# Independent Interactions of Phosphorylated β-Catenin with E-Cadherin at Cell-Cell Contacts and APC at Cell Protrusions

**DOI:** 10.1371/journal.pone.0014127

**Published:** 2010-11-30

**Authors:** Maree C. Faux, Janine L. Coates, Nadia J. Kershaw, Meredith J. Layton, Antony W. Burgess

**Affiliations:** 1 Ludwig Institute for Cancer Research, Melbourne, Victoria, Australia; 2 The Walter and Eliza Hall Institute of Medical Research, Melbourne, Victoria, Australia; 3 The Department of Biochemistry and Molecular Biology, Monash University, Melbourne, Victoria, Australia; University of Washington, United States of America

## Abstract

**Background:**

The APC tumour suppressor functions in several cellular processes including the regulation of β-catenin in Wnt signalling and in cell adhesion and migration.

**Findings:**

In this study, we establish that in epithelial cells N-terminally phosphorylated β-catenin specifically localises to several subcellular sites including cell-cell contacts and the ends of cell protrusions. N-terminally phosphorylated β-catenin associates with E-cadherin at adherens junctions and with APC in cell protrusions. We isolated APC-rich protrusions from stimulated cells and detected β-catenin, GSK3β and CK1α, but not axin. The APC/phospho-β-catenin complex in cell protrusions appears to be distinct from the APC/axin/β-catenin destruction complex. GSK3β phosphorylates the APC-associated population of β-catenin, but not the cell junction population. β-catenin associated with APC is rapidly phosphorylated and dephosphorylated. HGF and wound-induced cell migration promote the localised accumulation of APC and phosphorylated β-catenin at the leading edge of migrating cells. APC siRNA and analysis of colon cancer cell lines show that functional APC is required for localised phospho-β-catenin accumulation in cell protrusions.

**Conclusions:**

We conclude that N-terminal phosphorylation of β-catenin does not necessarily lead to its degradation but instead marks distinct functions, such as cell migration and/or adhesion processes. Localised regulation of APC-phospho-β-catenin complexes may contribute to the tumour suppressor activity of APC.

## Introduction

Mutation of the *Adenomatous Polyposis Coli (APC)* gene is responsible for over 80% of colon tumours, and is the first detectable event in colorectal neoplasia [1], [2]. The current paradigm for most colon cancers is that truncation of APC disrupts the regulation of the cellular concentration of β-catenin by proteasome-mediated degradation [3], [4]. The critical step is the phosphorylation of β-catenin at Ser33 and Ser37, within an APC- and axin-containing complex known as the “β-catenin destruction complex”. PhosphoSer33/37-β-catenin comprise a recognition sequence for the βTrCP E3 ligase, resulting in the ubiquitination and subsequent degradation of β-catenin [5], [6], [7]. Mutations in β-catenin in a number of tumour types can also prevent its phosphorylation [8]. The failure of the APC-axin-β-catenin destruction complex to phosphorylate β-catenin is proposed to result in the accumulation of β-catenin and subsequent constitutive activation of Wnt/β-catenin-target genes, which are important in gut development and in driving colorectal tumourigenesis [9], [10], [11].

Evidence is accumulating that the mechanism of regulation of different cellular processes by β-catenin is more complex than simply involving the control of total cellular levels of the protein [12], [13], [14]. β-catenin has been proposed to be regulated by changes in its conformation, where different molecular forms act in adhesion or transcription, respectively [14]. β-catenin is also regulated by E-cadherin-mediated cell-cell adhesion and tyrosine phosphorylation [13], [15]. For example, phosphorylation of β-catenin at Tyr654 results in its dissociation from E-cadherin [16] and phosphorylation at Tyr142 acts as a switch that disrupts its binding to α-catenin and promotes binding of BCL9-2, a nuclear co-factor, resulting in increased transcription of Wnt-target genes [13].

While several studies using phospho (Ser33/37/41/45)-specific β-catenin antibodies reinforce the concept that N-terminal phosphorylation of β-catenin leads to its degradation [17], [18], recent work implicates N-terminally phosphorylated β-catenin in distinct functions, including microtubule regrowth at centrosomes [19] and Wnt5a-induced β-catenin/E-cadherin association that does not lead to β-catenin degradation [20]. More recently, phosphorylation of β-catenin at Ser45 has been reported to be spatially separate from phosphorylation at Ser33/37/41 and appears to be involved in a distinct nuclear function [21]. These studies raise the prospect that N-terminal phosphorylation of β-catenin may not necessarily lead directly to its degradation.

In addition to regulation of β-catenin degradation, APC has been implicated in a range of cellular processes based on interactions with cytoskeletal proteins [22], [23]. Several of these functions are altered by cancer-associated *APC* mutations [22]. However, little is known about the mechanisms by which the different interactions (and functions) of APC are regulated. Recently, phosphorylated APC and β-catenin were reported at microtubule-dependent clusters and phosphorylated APC proposed to play a role in directional migration independent of β-catenin transcriptional activity or cell-cell adhesion [24]. In the present study, we establish that N-terminally phosphorylated β-catenin is localised to distinct subcellular sites in epithelial cells, including Ca^2+^-dependent cell-cell contacts and with APC in clusters at microtubule ends in cell protrusions. Here, β-catenin associated with APC exists in a complex that is separate from axin and is rapidly phosphorylated and dephosphorylated by a GSK3β-dependent mechanism. Localised phospho-β-catenin/APC clusters accumulate in migrating cells and are lost in colon cancer cells when APC is truncated. The subpopulation of phospho-β-catenin at the microtubule tips appears to play a role in the tumour suppressor function of APC.

## Results

### β-catenin is phosphorylated at distinct subcellular sites in epithelial cells

**Figure 1:**
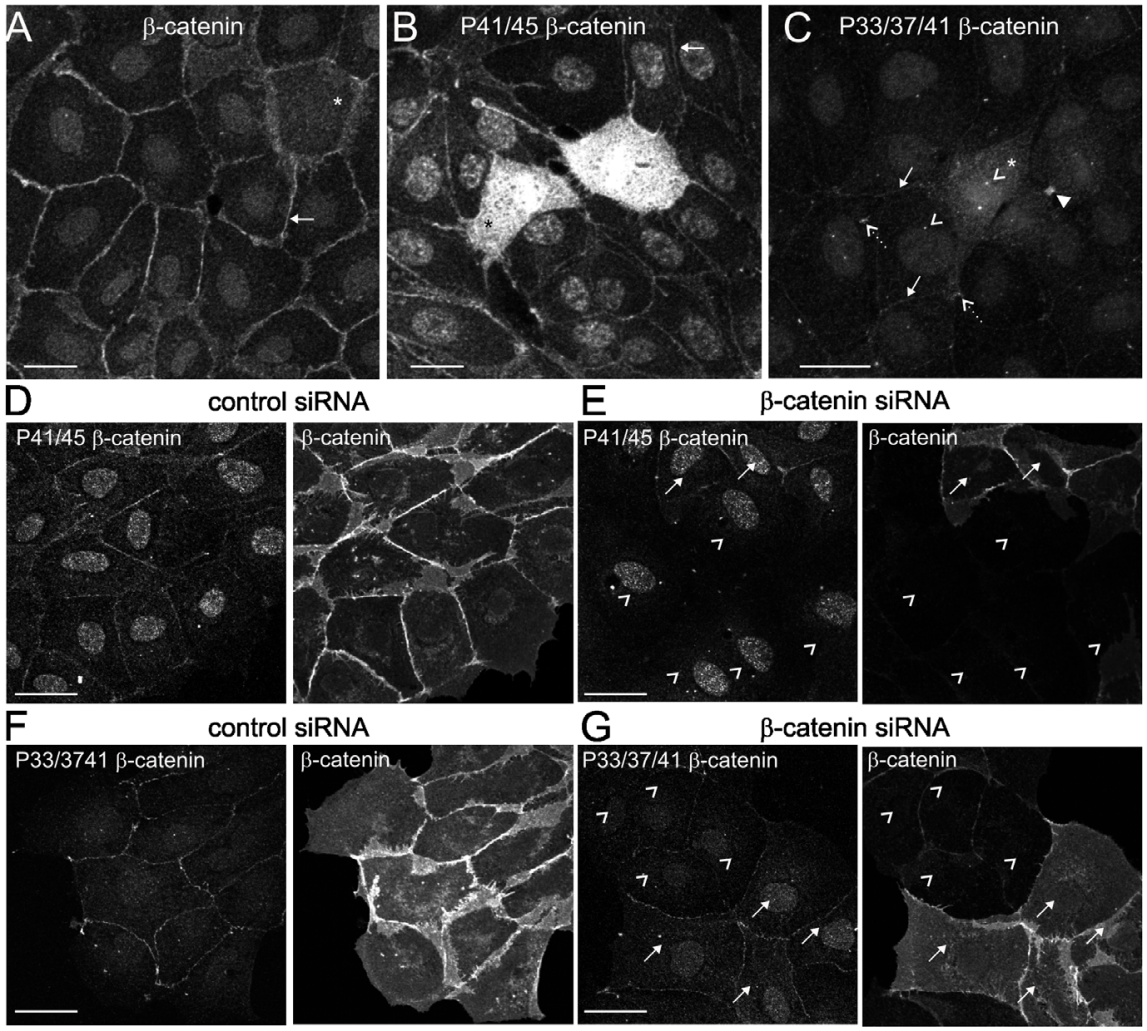
Populations of phosphorylated β-catenin in epithelial cells. (**A**) β-catenin is localised to cell-cell contacts (arrow). Mitotic cell is indicated by *. (**B**) Phospho-β-catenin pT41/pT45 (P41/45) is localised to the cell membrane (arrow) and mitotic cell cytoplasm (*). (**C**) Phospho β-catenin pS33/pS37/pT41 (P33/37/41) is localised to the cell membrane (arrows), in discrete clusters (dashed arrows) and the mitotic cell cytoplasm (*). The staining at centrosomes (open arrowheads) and mid-body bridges (filled arrowhead) is indicated. (**D–G**) MDCK cells were treated with siRNA directed against β-catenin or a control sequence. Cells were fixed 72 h after siRNA transfection and immunostained with antibodies to P41/45 β-catenin and β-catenin (**D and E**) and P33/37/41 β-catenin and β-catenin (**F and G**). Fields of cells without knockdown (arrows) are shown adjacent to β-catenin depleted cells (arrowheads) for comparison. Scale bars 20 µm.

To investigate β-catenin phosphorylation, we examined the localisation of β-catenin in Madin-Darby Canine Kidney 3 (MDCK) epithelial cells using two phospho-specific β-catenin antibodies. One antibody was raised against β-catenin phosphorylated on Thr41 and Ser45 [P41/45] and the other was raised against phosphorylation at Ser33, Ser37 and Thr41 [P33/37/41]. We first confirmed that β-catenin is predominantly localised to cell-cell contacts in MDCK epithelial monolayers using a pan β-catenin antibody that recognises all molecular forms of β-catenin [25] (Layton et al, submitted) (**Fig. 1A**). β-catenin is reported to be phosphorylated first on Ser45 by CK1α which creates a priming site for GSK3β to subsequently phosphorylate Thr41, Ser37 and Ser33 [26], [27]. Despite phosphorylation at Ser33 and Ser37 having the potential to target β-catenin for destruction [6], [28], [29], stable populations of phospho-β-catenin recognised by either P41/45 or P33/37/41 phospho-specific antibodies are found at distinct subcellular sites, including cell-cell junctions, the mitotic cell cytoplasm and discrete clusters (**Fig. 1B and C**). The P33/37/41-phospho-β-catenin antibodies also detect staining at centrosomes and midbody bridges in mitotic cells and in the nucleus (**Fig. 1B and C**). In contrast to previous studies [17] phospho-β-catenin was detected in the absence of treatments that block the proteasome.

To test the specificity of P41/45- and P33/37/41-phospho-β-catenin antibodies for the different subpopulations, four control experiments were employed. Firstly, β-catenin was depleted by siRNA (∼70% reduction; **Fig S1**). Immunofluorescent staining by P41/45- and P33/37/41-β-catenin antibodies at cell-cell contacts and in clusters was markedly reduced in β-catenin depleted cells (**Fig. 1** **, D–G**), indicating that these populations represent phospho-β-catenin, although the nuclear signal remained, suggesting that it is not specific for phospho-β-catenin. Secondly, competition with phospho-peptides of the same sequence as the antigen used to raise phospho-β-catenin antibodies, competed for detection of distinct subcellular populations by P41/45- and P33/37/41-β-catenin, with only the nuclear signal remaining, again suggesting that the nuclear staining may be non-specific (**Fig. S2**). Thirdly, phosphorylation of recombinant β-catenin *in vitro* demonstrates that P41/45 antibodies recognise β-catenin phosphorylated by CK1α alone or CK1α plus GSK3β; P33/37/41 antibodies recognise β-catenin phosphorylated by CK1α plus GSK3β but not by CK1α nor GSK3β alone (**Fig. S3**). This data confirms that CK1α phosphorylates Ser45 while GSK3β is required for phosphorylation of Thr41, Ser37, Ser33. In addition, phosphorylation of Thr41 by GSK3β is not necessary for recognition by the P41/45 antibody, suggesting that it predominantly recognises phospho-Ser45. Neither antibody recognises unphosphorylated β-catenin (**Fig. S3**). Finally, neither P41/45- nor P33/37/41-β-catenin antibodies detect β-catenin in interphase LIM2551 colon cancer cells that harbour a mutation resulting in deletion of the epitope for the phospho-β-catenin antibodies (β-catenin residues 5–72 [30]) (**Fig. S4A and Fig. S5**). We note that the P33/37/41 phospho-β-catenin antibodies also stain centrosomes and mid-body bridges in mitotic LIM2551 cells (**Fig. S4B**), indicating that the apparent localisation of phospho-β-catenin at centrosomes and concentrated at the mid-body during mitosis [19], [31] (see Fig. 1C, arrowheads) is not specific for phospho-β-catenin.

We examined MDCK and 6 colon cancer cell lines and found that the major band detected by P41/45- and P33/37/41-phospho-β-catenin antibodies co-migrates with β-catenin (**Fig. S5**). Cells containing mutations in APC (LIM2537 and SW480) have detectable phospho-β-catenin whereas cells containing β-catenin mutations at Ser45 (LIM1899 and HCT116) do not contain phospho-β-catenin recognised by P41/45. However, the P41/45 phospho-β-catenin antibody still recognises T41A β-catenin (LIM1215) further demonstrating that the P41/45 antibody primarily recognises phospho-Ser45 rather than phospho-Thr41. Interestingly, Ser45 mutant β-catenin, which lacks the ability to be ‘primed’ for GSK3β phosphorylation by CK1α, is still recognised by P33/37/41 (**Fig. S5**), consistent with previous findings [32], underscoring the complexity of β-catenin regulation. As expected, inhibition of GSK3β with LiCl did not show a change in P41/45-β-catenin but does reduce the P33/37/41 signal (**Fig. S5**). These data suggest that stable populations of N-terminally phosphorylated β-catenin are associated with different subcellular sites, and that, in contrast to prevailing models, β-catenin phosphorylation may not only be involved in degradation.

### Phosphorylated-β-catenin localised to cell-cell contacts requires cell-cell adhesion

**Figure 2:**
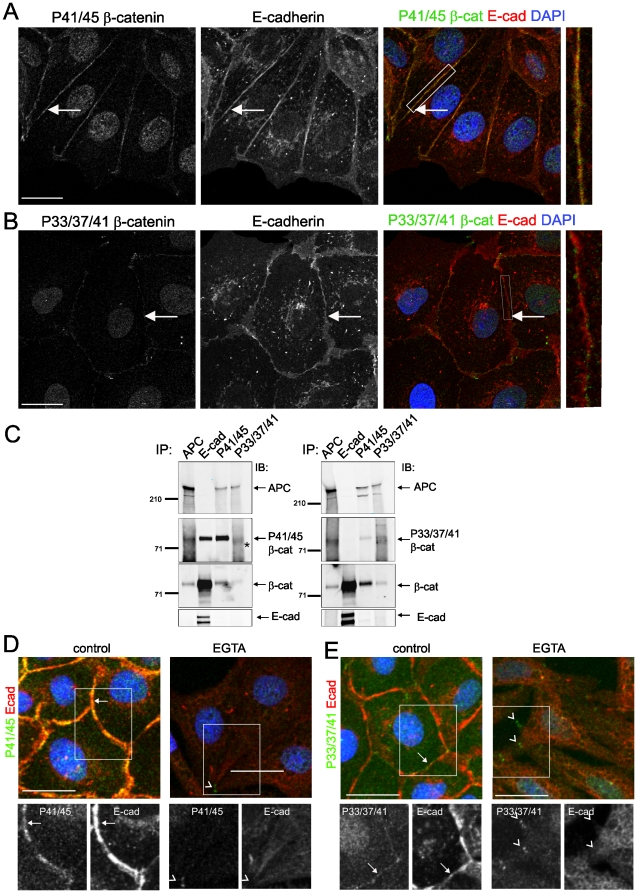
Phosphorylated β-catenin associates with E-cadherin at cell-cell adherens junctions. (**A**) P41/45-β-catenin is localised to cell-cell contacts with E-cadherin. (**B**) P33/37/41-β-catenin is localised to the cell periphery and shows partial overlap with E-cadherin. Cell contacts are indicated by arrows. Inset, enlarged view of overlapping phospho-β-catenin (green) and E-cadherin (red). P41/45-β-catenin (**A**) shows more prominent cell contact staining and overlap with E-cadherin than P33/37/41-β-catenin (**B**). (**C**) Co-precipitation of phospho-β-catenin with E-cadherin and APC in MDCK cells. APC, E-cadherin, P41/45- and P33/37/41-β-catenin immunoprecipitates were immuno-blotted with the indicated antibodies. * denotes non-specific band. (**D and E**) Peripheral phospho-β-catenin is regulated by Ca^2+^-dependent cell-cell contact. Cell contact associated E-cadherin and phospho-β-catenin staining is intact in control cells (arrows), but disrupted in treated cells where phospho β-catenin clusters remain (arrowheads). Phospho-β-catenin (green), E-cadherin (red), nuclei (DAPI) blue. Shown are representatives of at least three different experiments; scale bars 20 µm.

We then examined which proteins are associated with populations of phospho-β-catenin in different subcellular locations. β-catenin phosphorylated at Thr41 and Ser45 (as detected by the P41/45 antibody) is localised to cell-cell contacts with E-cadherin (**Fig. 2A**). Peripheral P33/37/41-β-catenin staining shows minimal overlap with E-cadherin (**Fig. 2B**, arrows). The peripheral phospho-β-catenin localisation is similar to partial co-localisation with E-cadherin reported previously [18]. E-cadherin co-precipitates P41/45-β-catenin whereas P33/37/41-β-catenin is not detected in the E-cadherin precipitate (**Fig. 2C**), suggesting that peripheral P33/37/41-β-catenin may not be associated with cell-cell adhesions. This may be due to low levels of P33/37/41-β-catenin and reflect that only a small proportion of total P33/37/41-phospho-β-catenin may be associated with E-cadherin.

We investigated the nature of peripheral phospho-β-catenin further by testing the impact of dissociation of calcium dependent adhesions. Chelation of extracellular calcium disrupts cell-cell contacts (**Fig. 2D and E**). Significantly, the peripheral populations of both P41/45- and P33/37/41-β-catenin are lost completely, whereas other populations of phospho-β-catenin, notably those in discrete clusters at the ends of cell protrusions, remain (**Fig. 2D and E**, arrowheads). These results suggest that localisation of phospho-β-catenin to the cell periphery requires intact calcium-dependent cell-cell adhesions. Furthermore, we do not detect axin at the sites of cell contact (**Fig. S6**), in contrast to Maher et al [18], suggesting that the peripheral, cadherin-associated phospho-β-catenin that we detect is not part of a destruction complex.

### Phosphorylated β-catenin localised with APC at microtubule ends does not require cell-cell adhesion

**Figure 3:**
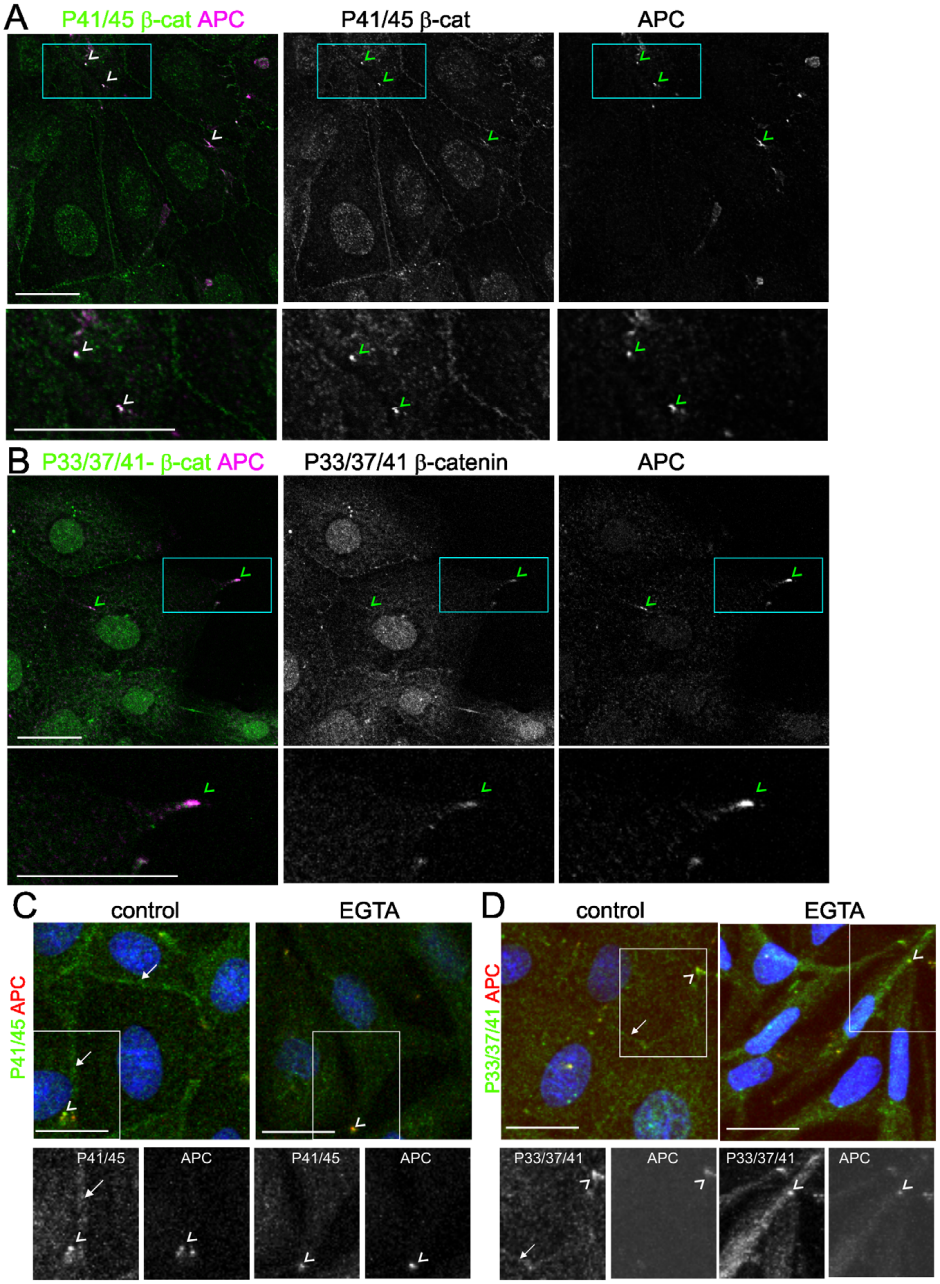
Phosphorylated β-catenin associates with APC at the ends of microtubules. (**A and B**) Phosphorylated β-catenin is localised in clusters with APC in MDCK cells. Coincident APC (magenta) and phospho-β-catenin (green) is indicated with arrowheads. (**C and D**) Disruption of Ca^2+^-dependent cell-cell contact does not alter phospho-β-catenin coincident with APC in clusters at cell extensions (arrowheads). Junctional phospho-β-catenin staining is intact in control cells (arrows). Phospho-β-catenin (green), APC (red), nuclei (DAPI) blue. Shown are representatives of at least three different experiments; scale bars 20 µm.

Co-staining with APC reveals that P41/45- and P33/37/41-β-catenin cluster with APC at the ends of microtubules (**Fig. 3A and B**, arrowheads, β-tubulin co-stain is shown in, **Fig. S7**). The clusters of P33/37/41-β-catenin staining in **Fig. 1C** (dashed arrows) are also coincident with APC (not shown). Note that the majority of APC decorates the ends of microtubules at cell protrusions in subconfluent epithelial cells, as previously reported [33].

Given that the phospho-β-catenin populations localised at cell-cell contacts with E-cadherin and at the ends of microtubules with APC are spatially separate, and that β-catenin binding to APC and E-cadherin is mutually exclusive [34], it is clear that the APC/phospho-β-catenin complex is distinct from the E-cadherin/phospho-β-catenin complex. E-cadherin co-precipitates P41/45-β-catenin but not APC; whereas APC co-precipitates P41/45- and P33/37/41-β-catenin, but not E-cadherin (**Fig. 2C**). Accordingly, the phospho-β-catenin coincident with APC at cell extensions remains after disruption of calcium-dependent cell-cell adhesion (**Fig. 3C and D**, arrowheads) whereas junctional E-cadherin/phospho-β-catenin is lost. This data suggests that the cell-cell contact population of phospho-β-catenin (predominantly P41/45) is associated with E-cadherin in calcium-dependent cell-cell adhesions and that there is a separate population of phospho-β-catenin associated with APC clustered at the ends of microtubules at cell protrusions.

### Isolation of APC-rich protrusions

**Figure 4:**
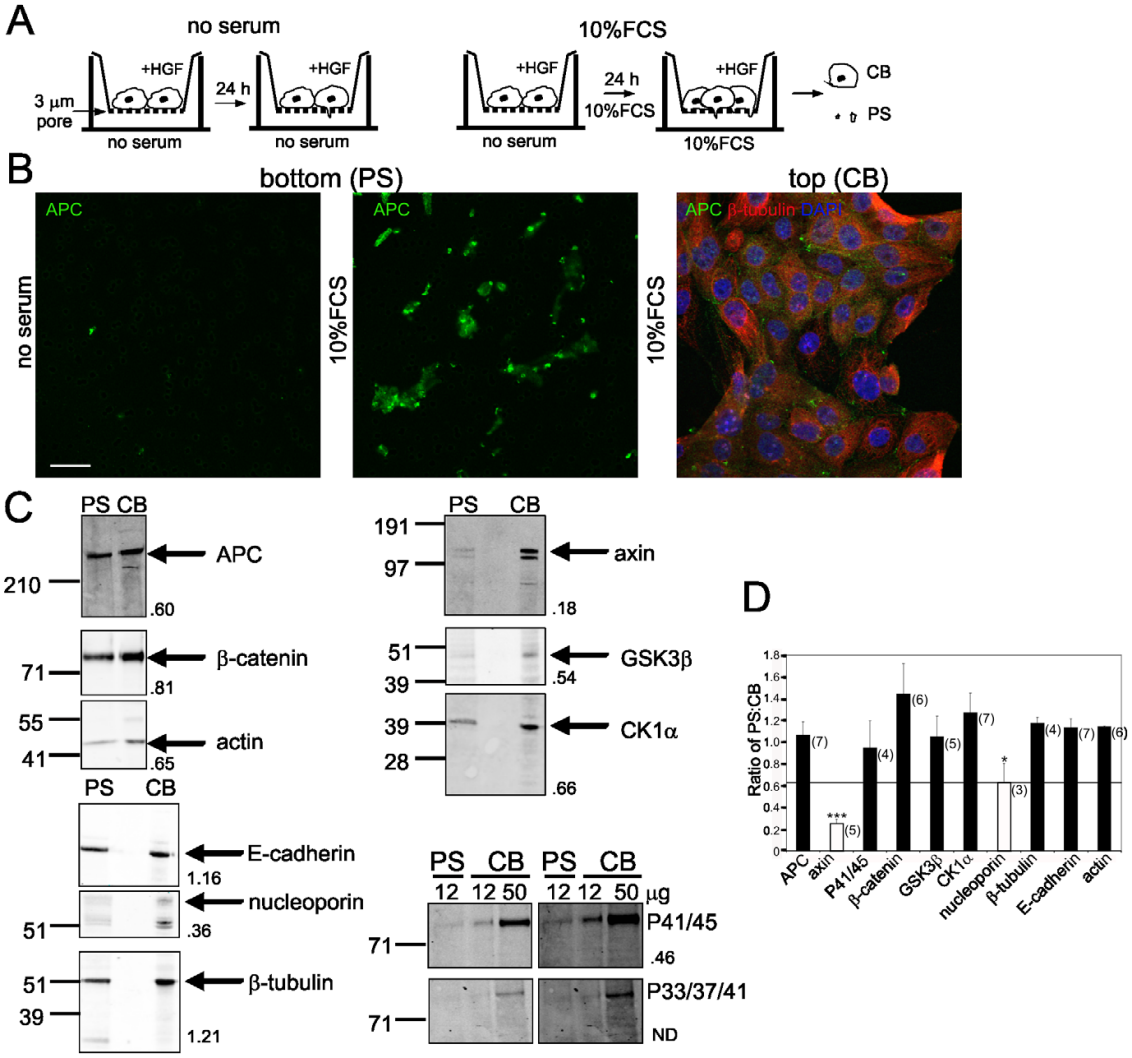
Isolation of APC from protruding pseduopodia. (**A**) Schematic diagram depicting strategy for isolation of pseudopodia. PS pseudopodia, CB cell bodies. (**B**) MDCK cells on 3.0 µm pore filters were induced to extend pseudopodial protrusions. Cells were fixed and either cell bodies were removed leaving pseudopodia, bottom (PS), or pseduopodia were removed leaving cell bodies, top (CB). Filters were immunostained for APC (green) and β-tubulin (red) and costained with DAPI (blue).Cells protrusions contain APC. DAPI staining confirms there are no nuclei on the lower surface, bottom (PS). Scale bar, 20 µm. (**C**) Isolation of APC and β-catenin, but not axin, from the pseudopodia (PS) fraction. Immunoblots of proteins isolated from the pseudopodia (PS) on the lower membrane surface or cell bodies (CB) on the upper membrane surface of 3.0 µm pore filters. Total protein loaded was 12 µg except for the P41/45 and P33/37/41 immunoblots where 50 µg CB was loaded in order to detect the proteins. The numbers to the side of blots are the normalised ratio of the amount of each protein in PS vs CB. Shown is representative from at least 3 independent experiments. (**D**) APC, but not axin, is present in the PS fraction. Proteins isolated from the PS or CB fractions were resolved by SDS-PAGE and immunoblotted as in (C). Shown is the average +/− SEM of the ratio of PS∶CB for the indicated protein from at least three separate experiments (n is indicated). ***P<0.002, *P<0.05 by Student's *t*-test.

The distinctive localisation of APC to the ends of microtubules is linked to a role in microtubule stabilisation and cell migration [35], [36]. We focussed on phospho-β-catenin coincident with APC clusters and sought to isolate APC-rich cell protrusions and examine the proteins in APC protein complexes. Cells were plated on a porous 3.0 µm membrane and induced to extend pseudopodial protrusions through the membrane in response to a stimulus [37]. We fractionated these cells into pseudopodia (PS) and cell body (CB) fractions (**Fig. 4A**). We established that the protrusions that extend past the lower surface of the membrane in response to serum stimulation are enriched for APC (**Fig. 4B**). In addition, nuclei are not present on the lower surface of the membrane but were detected only in the cell bodies (**Fig. 4B**). In order to isolate sufficient pseudopodial proteins, cells were plated on the filters at high density. Accordingly, a significant proportion of cells were not able to extend protrusions to the underside of the filter, and some APC clusters at the ends of microtubules are still present on the upper surface of the membrane (**Fig. 4B**). These findings indicate that although APC is present at the microtubule tips on both the upper and lower surfaces of the filter, extraction of pseudopodia from the lower surface provides a means to biochemically isolate the APC that is concentrated in protrusions.

We confirmed that APC is present in pseudopodia and cell bodies by immunoblotting isolated CB and PS fractions (**Fig. 4C, D**). In the absence of serum, little APC, as well as β-tubulin, is detected in the pseudopodia fraction (data not shown), consistent with immunofluorescent staining (**Fig. 4B**) and indicating that serum is required for pseudopodia formation and/or extension. The isolated pseudopodia fraction contained only low level contamination of proteins exclusive to the cell body. No nuclear staining (DAPI) was apparent in the pseudopodia (**Fig. 4B**) therefore no nucleoporin, a nuclear membrane protein, should appear in the PS fraction (**Fig. 4C, D**). The contribution of CB proteins to the PS fraction is therefore defined by the proportion of nucleoporin in the PS (**Fig. 4D**). Proteins with a greater proportion in the PS versus the CB fraction compared to nucleoporin are therefore enriched in pseudopodia or distributed similarly between the two fractions. Importantly, proteins with a lower proportion in the PS compared to nucleoporin must be excluded from the pseudopodia.

### APC/β-catenin in cell protrusions is distinct from the APC/β-catenin/axin complex

A significant proportion of total β-catenin is present in the pseudopodia, together with E-cadherin (**Fig. 4C, D**). E-cadherin and β-catenin are also detected in the PS fraction by immunofluorescence (not shown). As expected, β-tubulin and actin are also present in pseudopodia (**Fig. 4C, D**). We next examined proteins shown to associate with APC and β-catenin in the ‘destruction complex’. Consistent with our previous findings [38], only a very small proportion of axin is present in the pseudopodia compared to the cell bodies (less than 4-fold compared to APC, P<0.002), suggesting that any axin:APC complex is spatially separate from APC concentrated in cell protrusions (**Fig. 4C, D**). Moreover, the proportion of axin in the PS fraction was consistently less than the proportion of nucleoporin in the PS fraction, indicating that any axin detected in this fraction must be due to low level CB contamination of the PS fraction (**Fig. 4D**). The proportion of APC, β-catenin, GSK3β and CK1α, as well as PP2A (not shown), detected in the PS fraction was consistently greater than that of nucleoporin (**Fig. 4C, D**), demonstrating that they are genuinely present, although not necessarily enriched in pseudopodia. Thus the APC/β-catenin complex in cell protrusions is distinct from the APC/axin/β-catenin destruction complex.

### β-catenin associated with APC at the ends of microtubules is phosphorylated by GSK3β

**Figure 5:**
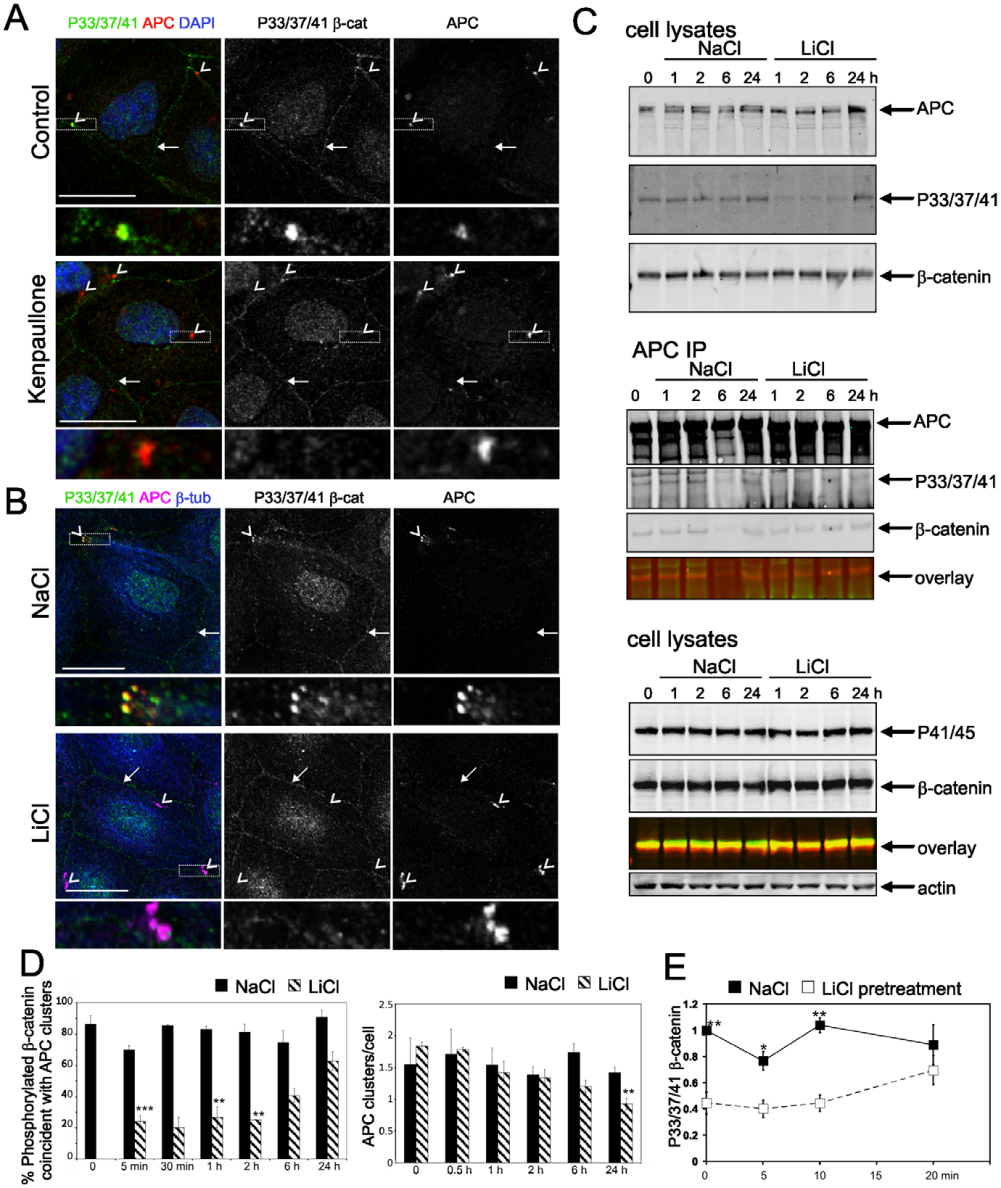
Inhibition of GSK3β results in loss of P33/37/41-β-catenin associated with APC but not other sub-populations. (**A**) Kenpaullone (2 nM, 30 min) and (**B**) LiCl (30 mM, 5 min) treatment of MDCK cells results in loss of P33/37/41-β-catenin coincident with APC. APC clusters (arrowheads) are coincident with P33/37/41-β-catenin in control but not kenpaullone or LiCl treated cells; intact peripheral staining is indicated by arrows. Scale bars 20 µm. (**C**) LiCl treatment results in reduced P33/37/41-β-catenin in total cell lysates and associated with APC, but not P41/45-β-catenin. Lysates from MDCK cells treated with 30 mM NaCl or LiCl for T0, 1, 2, 6 and 24 h were immunoblotted or immunoprecipitated with APC antibodies and immunoblotted as indicated. Overlay refers to the merge of P33/37/41 or P41/45-anti-rabbit-IR800 and β-catenin-anti-mouse-IR680 channels. (**D**) Analysis of coincident P33/37/41-β-catenin and APC clusters following GSK3β inhibition. MDCK cells were treated with 30 mM NaCl or LiCl for the indicated times, fixed and immunostained. Cells were scored for coincident P33/37/41-β-catenin and APC clusters (left) and number of APC clusters/cell (right). Shown is average +/− SEM from three (T0, 5 min, 1, 2 6 and 24 h) or two (T30 min) independent experiments, >100 cells per treatment group; ***P<0.001, **P<0.01 by Student's *t*-test. (**E**) Removal of GSK3β inhibition allows re-phosphorylation of β-catenin. MDCK cells pretreated with 30 mM NaCl or LiCl for 30 min, were incubated in DME+10%FCS and harvested at T0, 5, 10 and 20 min. Lysates were immunoblotted with P33/37/41 β-catenin and actin antibodies. Shown is average +/− SEM from four independent experiments for normalised densitometry values, *P<0.05, **P<0.01 by Student's *t*-test. A representative blot is shown in Fig S10A.

To test whether the different subpopulations of N-terminally phosphorylated β-catenin are GSK3β-dependent, we treated cells with two different GSK3β inhibitors. Both kenpaullone and LiCl treatment results in loss of APC-coincident P33/37/41 β-catenin clusters (**Fig. 5A and B**) indicating that β-catenin is phosphorylated by GSK3β at this site. Reduction in P33/37/41 β-catenin following kenpaullone and LiCl treatment was confirmed by immunoblot analysis (**Fig. S8A**). P41/45 β-catenin is not affected by GSK3β inhibitors, as expected (**Fig. S8B and C**). The localisation of P41/45 β-catenin with APC clusters implies that phosphorylation at Ser33, Ser37 and Thr41 by GSK3β does not result in recruitment of β-catenin to APC clusters, but instead that β-catenin resident in these complexes is phosphorylated by GSK3β *in situ*. Surprisingly, the P33/37/41 β-catenin at cell contacts is not affected by inhibition of GSK3β even after long treatments (**Fig. 5A and B**) suggesting that GSK3β is not involved in phosphorylating these populations of β-catenin. Both P33/37/41- and P41/45-β-catenin at cell-cell contacts are reduced following treatment with CK1 inhibitor, D4476 (**Fig. S8A and B; Fig. S9**). The loss of P33/37/41- and P41/45-β-catenin cell contact staining upon CK1 but not GSK3β inhibition (**Fig. S9**) indicates that CK1, but not GSK3β, is involved in β-catenin phosphorylation at cell-cell adhesions. This implies that either CK1 phosphorylates P33/37/41 localised at the cell periphery or that S45 phosphorylation is required for these sites to be phosphorylated by another priming-dependent kinase. Thus, β-catenin is phosphorylated both by CK1 and GSK3β, but is selectively phosphorylated by different kinases at different sub-cellular sites.

In astrocytes, GSK3β has been reported to be specifically inactivated in cell protrusions, leading to the association of APC with the plus ends of microtubules [39]. Accumulation of β-catenin at the leading edge in these cells is attributed to the localised inhibition of GSK3β. Although our observations suggest that APC localisation to the ends of microtubules is unaffected by inhibition of GSK3β and that the β-catenin which accumulates with APC is phosphorylated by GSK3β, and is unaffected by degradation, it is possible that these are separate events and that examining total β-catenin masks the phospho-specific events. In addition, the rate of phosphorylation and the incubation period for inhibitors may be crucial. The role of GSK3β is complex and short term LiCl treatment (up to 4 h) has been reported to result in dramatically different effects on β-catenin compared to longer term inhibition (>6 h) (Gottardi and Gumbiner, 2004).

Consequently, we examined the kinetics of GSK3β-dependent phosphorylation of localised β-catenin in lysates from control or LiCl treated cells. Inhibition of GSK3β for one hour results in reduced P33/37/41-β-catenin and this persists for several hours with LiCl treatment before phosphorylation begins to be restored (**Fig. 5C**). The P33/37/41-β-catenin co-precipitated with APC is also reduced in GSK3β inhibited cells compared to control, whereas total β-catenin is similar in control and treated cells (**Fig. 5C**). P41/45-β-catenin is not altered in LiCl-treated cells (**Fig. 5C** **and Fig. S8**), which suggests that the loss of phospho-β-catenin upon GSK3β-inhibition is not due to protein turnover/degradation. P33/37/41-β-catenin coincident with APC at the ends of microtubules is reduced within 5 min of LiCl treatment and is depleted for several hours (**Fig. 5D**), consistent with immunoblot analysis of total lysates and APC immunoprecipitates (**Fig. 5C**). The APC clusters themselves were not affected by Lithium treatment, although the number of clusters per cell was reduced with longer treatments (>6 h) (**Fig. 5D**). These data show that β-catenin localised with APC at the ends of microtubules is phosphorylated by GSK3β and that inhibition of GSK3β or CK1 does not have a marked effect on APC clusters.

### β-catenin associated with APC is phosphorylated and dephosphorylated rapidly

To investigate phosphorylation of β-catenin by GSK3β further, we first inhibited GSK3β by pretreatment with LiCl and then washed out the inhibitor and assessed P33/37/41-β-catenin levels. Consistent with rapid phosphorylation, P33/37/41-β-catenin increases after removal of LiCl from the media (**Fig. 5E** **, Fig. S10A**). P33/37/41-β-catenin co-incident with APC is also increased (**Fig. S10C** (P<0.02)). The rapid loss of phosphorylation upon treatment with LiCl indicates that a phosphatase may directly regulate β-catenin, as suggested in earlier studies [17]. While we observe only modest increases in P33/37/41-β-catenin in total cell lysates upon incubation with phosphatase inhibitors (**Fig. S10B**), there is a robust increase in the proportion of P33/37/41-β-catenin co-incident with APC (**Fig. S10C** (P<0.0001)). Interpretation of these experiments is complicated by any direct effects of phosphatase inhibitors and LiCl treatment on the auto-inhibitory Ser9 phosphorylation of GSK3β as has been reported [40], however, these data suggest that the phospho-β-catenin localised at the ends of microtubules is also regulated by phosphatases. Together, these data suggest that phospho-β-catenin observed in clusters at cell protrusions is associated with APC and constantly phosphorylated by GSK3β, and that other subpopulations of phospho-β-catenin at other subcellular sites are likely to be phosphorylated by other kinases.

### Phospho-β-catenin localisation is prominent at the ends of microtubules in migrating cells

**Figure 6:**
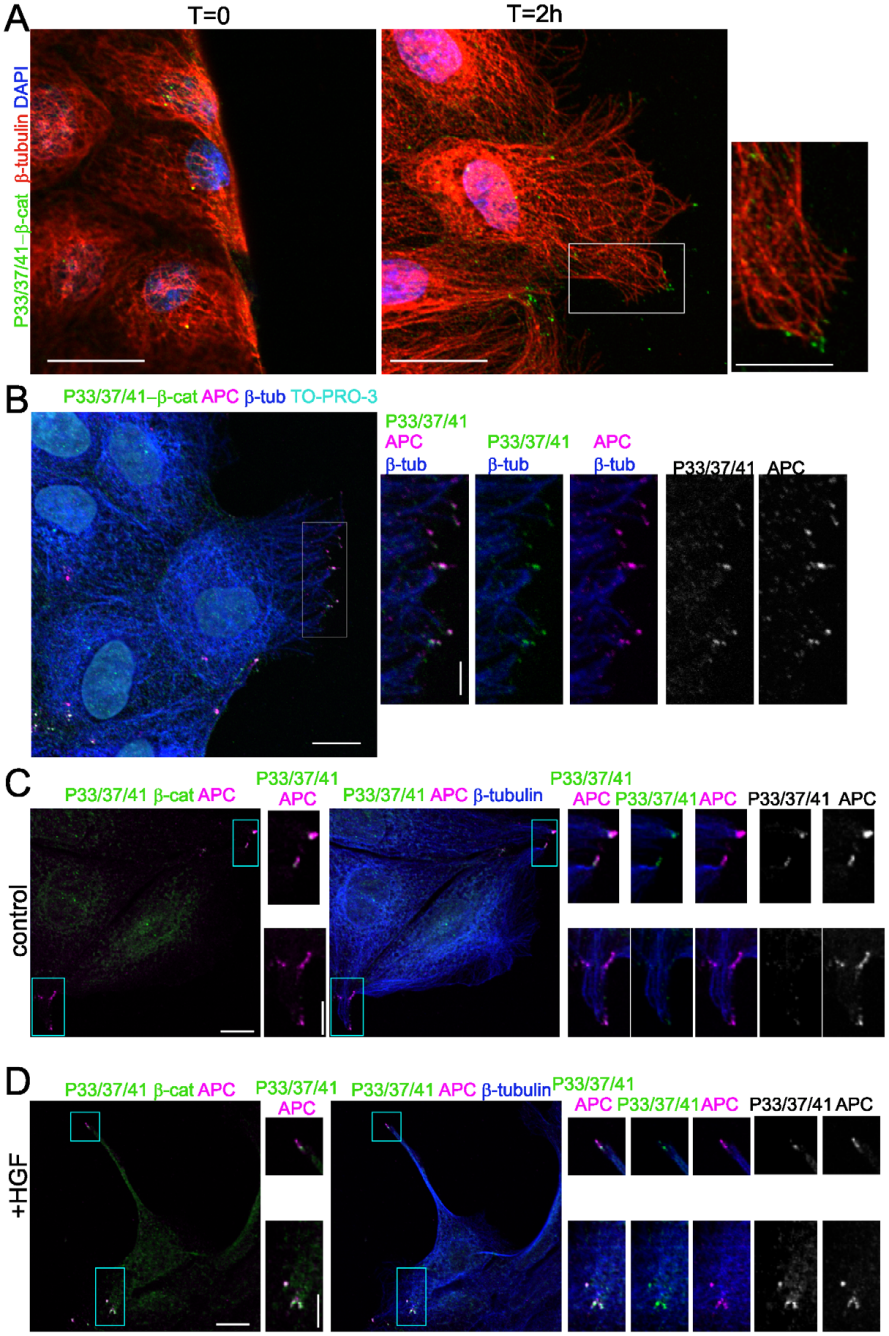
Phospho-β-catenin and APC are localised to the ends of microtubules in migrating cells. (**A**) P33/37/41-β-catenin in clusters at the wound edge. Representative images of cells fixed at T = 0 (left) and 2 hours after wounding. P33/37/41-β-catenin (green), β-tubulin (red), DAPI (blue). Scale 20 µm; inset 10 µm. (**B**) P33/37/41-β-catenin and APC in migrating MDCK cells. Merged image shows coincident (white) P33/37/41-β-catenin (green) and APC (magenta) at the ends of microtubules (β-tubulin, blue). Nuclei were visualised with TO-PRO-3 iodide staining (cyan). Scale 10 µm. Insets show enlarged view of the leading edge of migrating cells. Scale bar 5 µm. (**C and D**) MDCK cells were serum starved and untreated (**C**) or stimulated with 2 ng/ml HGF for 6 h (**D**) and immunostained with antibodies to P33/37/41-β-catenin (green), APC (magenta) and β-tubulin (blue). Scale 10 µm. Insets show clusters at microtubule ends that are highly concentrated in HGF treated cells. Scale bar 5 µm.

Given that APC localised to the plus ends of microtubules is implicated in microtubule dynamics and cell migration [36], [41], [42], we examined phospho-β-catenin in cells stimulated to migrate in a scratch wound assay. In subconfluent MDCK cells, APC is localised to the ends of a subset of microtubules, often in cell protrusions ([36], [42], see also **Fig. 3B**). One to two hours after wound-induced migration, P33/37/41-β-catenin accumulates in clusters at the leading edge of migrating cells (**Fig. 6A**) where APC also decorates the ends of microtubules (**Fig. 6B**). Importantly, P33/37/41-β-catenin accumulates with APC at the leading edge of migrating cells (**Fig. 6B**).

The accumulation of phospho-β-catenin in association with APC in migrating cells prompted us to examine the location of P33/37/41-β-catenin in cells stimulated with HGF. HGF stimulates epithelial cell motility, inducing disruption of cell-cell junctions and subsequent cell scattering [43]. In control cells, P33/37/41-β-catenin is concentrated at the ends of microtubules coincident with APC (**Fig. 6C**). HGF stimulation results in extended protrusion formation and prominent P33/37/41 β-catenin and APC staining at the ends of microtubules in these protrusions (**Fig. 6D**). Thus, induction of cell migration promotes the accumulation of phospho-β-catenin and APC at the leading edge. Whether phosphorylation of β-catenin at the ends of microtubules contributes to the process of cell migration or whether this occurs as a result of cell migration remains to be determined. Interestingly, phosphorylation of β-catenin at the microtubule ends in migrating epithelial cells contrasts with that observed in migrating astrocytes where GSK3β is inactivated at the leading edge [39].

### Phospho-β-catenin clusters localised to the ends of microtubules is dependent on APC

**Figure 7:**
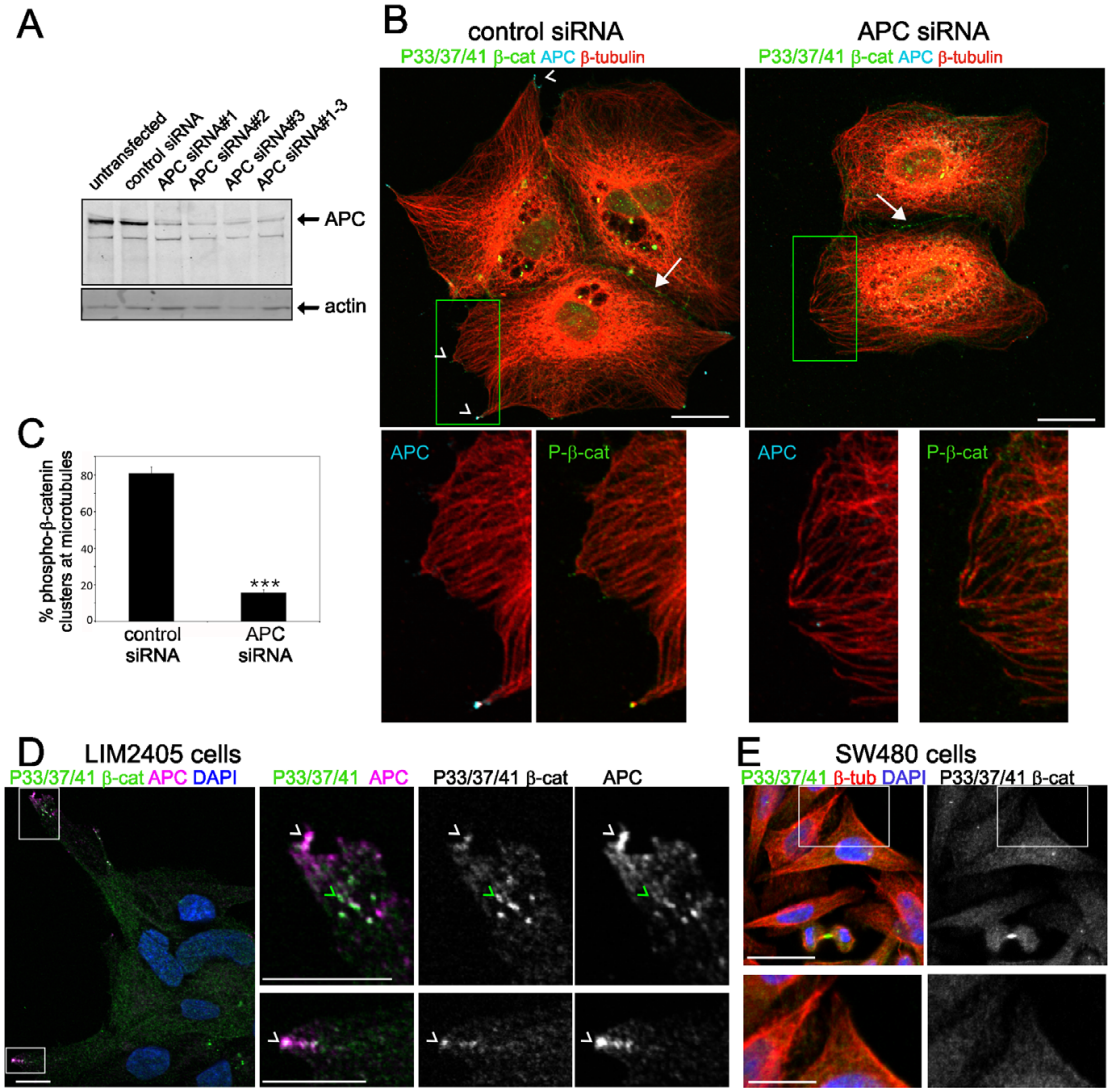
APC is required for phospho-β-catenin localisation to the ends of microtubules. (**A**) Depletion of cellular APC by siRNA. APC levels following siRNA transfection were assessed by immunoblot analysis in cell lysates. Actin was used as a loading control. (**B**) MDCK cells transfected with control and APC siRNA were immunostained for P33/37/41-β-catenin (green), APC (cyan) and β-tubulin (red) with APC/β-tubulin and P33/37/41-β-catenin/β-tubulin enlarged in insets shown below. Arrowheads indicate clusters of phospho-β-catenin at microtubule ends coincident with APC in control but not APC depleted cells. Scale bars 20 µm. (**C**) Quantification of phospho-β-catenin clusters (%) at microtubule protrusions in control and APC siRNA treated cells. Shown is average +/− SD from 3 independent experiments, >70 microtubule protrusions per treatment group. ***P<0.001 by Student's *t*-test. (**D**) LIM2405 colon cancer cells containing a wt APC allele demonstrate concentrations of P33/37/41-β-catenin (green) at cell extensions that partially overlap with APC (magenta) (white arrowheads indicate overlap with APC, green arrowheads indicate concentrations of P33/37/41-β-catenin that do not overlap with APC). Nuclei were visualised with DAPI; scale bars 10 µm. (**E**) P33/37/41-β-catenin is diffusely cytoplasmic in SW480 colon cancer cells and does not concentrate in clusters at microtubule ends. SW480 cells were immunostained with P33/37/41-β-catenin (green) and β-tubulin (red) and costained with DAPI to visualise nuclei, scale bar 20 µm. P33/37/41/β-tubulin and P33/37/41-β-catenin enlarged in insets are shown below, scale bar 10 µm.

We then tested whether loss of APC impacts phospho-β-catenin localised to the ends of microtubules by depleting APC with siRNA. Transfection of MDCK cells with siRNAs targeting APC reduces the level of APC (**Fig. 7A**) and results in loss of detectable P33/37/41-β-catenin clusters at the ends of microtubules (**Fig. 7B**). Quantification of phospho-β-catenin at microtubule protusions revealed a significant reduction in phospho-β-catenin clusters in APC siRNA treated cells compared to control (P<0.001) (**Fig. 7C**). E-cadherin staining defines the junctional signal in MDCK colonies and shows that concentrations of P33/37/41-β-catenin coincident with E-cadherin are unaffected in APC siRNA treated cells (**Fig. S11**, green arrows). Note that these accumulations of P33/37/41 are not coincident with APC.

To test the significance of APC in phospho-β-catenin localisation at cell extensions, we examined P33/37/41-β-catenin in colon cancer cells containing either intact or mutated APC. LIM2405 cells contain a single intact APC allele and harbour a mutation in the other allele which results in truncation at residue 2198 [30]. LIM2405 cells form loosely adherent colonies with the cells on the outer edges extending prominent protrusions that are decorated at the very ends by APC (**Fig. 7D**). P33/37/41-β-catenin also accumulates at the ends of these protrusions (**Fig. 7D**, white arrowheads) and is also frequently found in concentrations immediately before the ends of the extension (**Fig. 7D**, green arrowheads). SW480 cells contain only truncated APC, and in contrast to cells with intact APC, P33/37/41-β-catenin is not detected at the ends of microtubules, but is instead distributed diffusely throughout the cytoplasm (**Fig. 7E**). Together these data show that intact APC is required for phospho-β-catenin localisation in clusters at the ends of microtubules protrusions.

## Discussion

**Figure 8:**
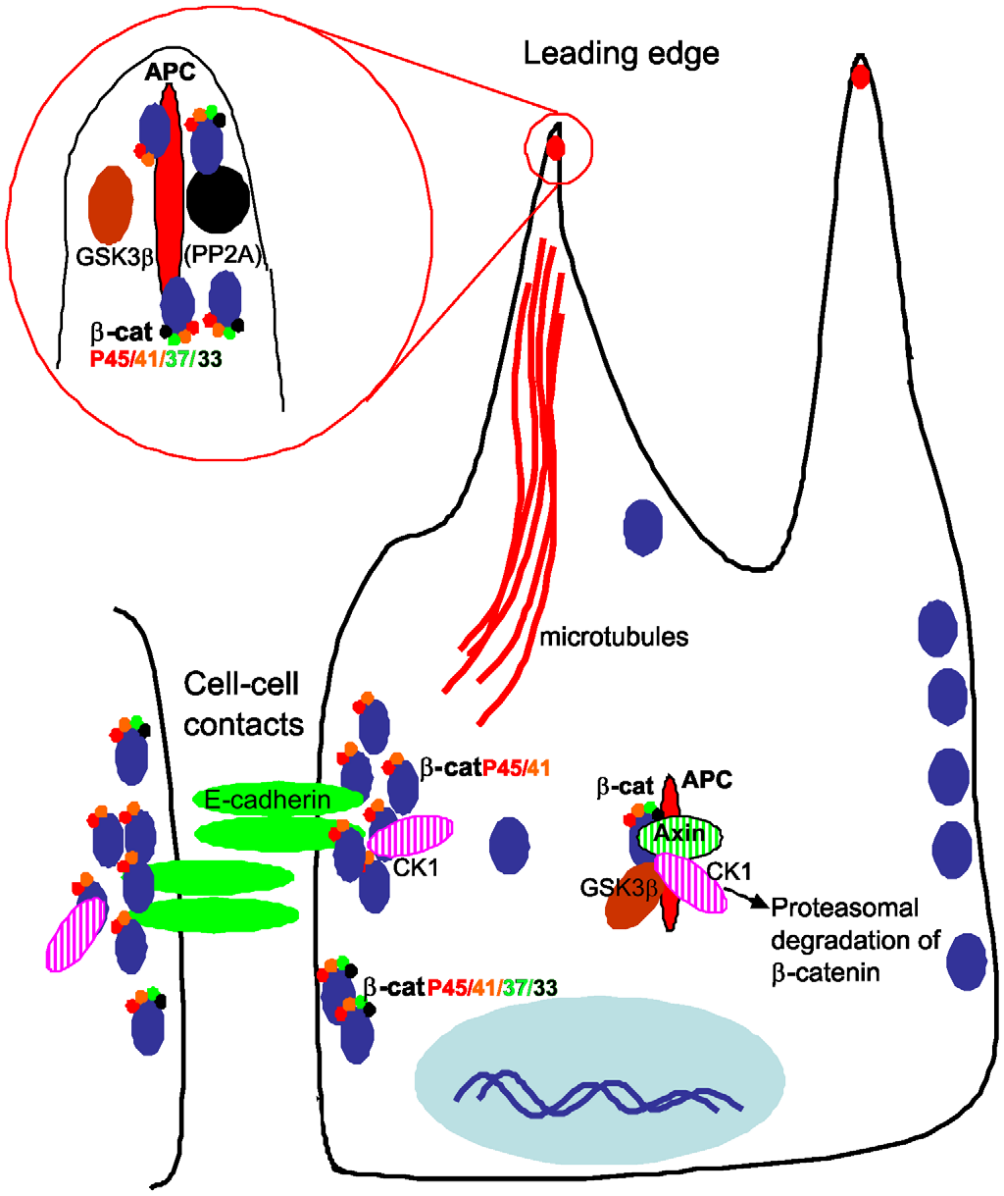
Functionally distinct populations of N-terminally phosphorylated β-catenin. P41/45-β-catenin associates with E-cadherin at cell-cell contacts. P33/37/41-β-catenin is also found at Ca^2+^-dependent cell contacts but is not detected in the complex with E-cadherin. P41/45- and P33/37/41-β-catenin associate with APC at the ends of microtubules at the leading edge of migrating cells. At the protrusions the phospho-β-catenin is rapidly phosphorylated and dephosphorylated. This complex is separate from the axin destruction complex that targets phospho-β-catenin for proteasomal degradation.

β-catenin is a critical component of cell-cell adhesions, where it forms a dynamic link between E-cadherin and the actin cytoskeleton [44], [45]. It is also a critical component of Wnt signalling, where it functions as a transcriptional activator of target genes that regulate cell proliferation and differentiation [15]. The canonical model of Wnt signalling requires that N-terminally phosphorylated β-catenin is targeted for destruction, thus providing a means for regulating the transcriptionally active protein. Our present findings establish that the different subcellular populations of N-terminally phosphorylated β-catenin are regulated quite differently and that phosphorylated β-catenin is not always targeted for destruction: 1. Phospho-β-catenin is detected without blocking the proteasome; 2. Mutation of S45 still results in detection of phospho-S33/S37/T41, as previously shown [32]; 3. Distinct subcellular locations of phospho-β-catenin implies participation in different functions; 4. Inhibition of GSK3β reduces the phospho-β-catenin at cell protrusions but does not appear to alter the junctional phospho-β-catenin populations; 5. A core component of the destruction complex, axin, is not present in cell protrusions with APC and is not detected at cell-cell junctions; 6. Accumulation of APC and phospho-β-catenin at the ends of cell protrusions and at the leading edge of migrating cells suggests that this population has an active role in microtubule regulation, cell-cell or cell matrix interactions. Our data suggest that localised phospho-β-catenin has distinct roles in normal epithelial cells (**Fig. 8**) and that the truncation of APC in many colon cancers would be expected to disturb these functions.

N-terminally phosphorylated β-catenin localised to sites of cell-cell contact is associated with E-cadherin and requires intact cell-cell adhesions. Phospho-β-catenin has recently been reported in two separate complexes: one with E-cadherin and the other with components of the destruction complex [18]. Cell-cell adhesion is proposed to influence the activity of the Wnt-signalling pool of β-catenin via a junction-localised phospho-destruction complex [18]. The loss of cell contact phospho-β-catenin that we observe following perturbation of Ca^2+^-dependent cell adhesion may reflect the increased transcriptionally active β-catenin reported by Maher et al [18]. However, we did not detect any change in the levels of total β-catenin (MCF and JLC, unpublished data), consistent with a recent report that also showed that the proteosomal inhibitor MG132 had little effect on β-catenin phosphorylation or Tcf/Lef-mediated transcription [24]. Significantly, we did not detect other destruction complex components (i.e. axin) at the cell periphery. Our results show that N-terminally phosphorylated β-catenin is not exclusively present in the destruction complex. Further, inhibition of GSK3β did not alter the cell-cell adhesion-associated populations of P41/45- or P33/37/41-β-catenin, indicating that this population of P33/37/41-β-catenin is likely to be phosphorylated by another kinase. This contrasts with the recent findings in endothelial cells that inhibition of GSK3β and CK1α results in reduced phospho-β-catenin at cell-cell contacts and in this cell type phospho-β-catenin was not detected in VE-cadherin immunoprecipitates [24]. The loss of cell contact phospho-β-catenin following CK1 inhibition implicates CK1 in the phosphorylation of β-catenin at Ser45 and possibly also Ser33/Ser37/Thr41. Alternatively, CK1 may be required to prime β-catenin at Ser45 for phosphorylation at Ser33/Ser37/Thr41 by another kinase. As recently proposed, phosphorylation at S45 may not only be important for priming β-catenin for subsequent phosphorylation [21]. Although P33/37/41-β-catenin is present at cell contacts in a Ca^2+^-dependent manner, it does not associate with E-cadherin by immunoprecipiation analysis, supporting the conclusion from Maher et al. [21] that P33/37/41-β-catenin can be uncoupled from P41/45. While tyrosine phosphorylation of β-catenin is reported to reduce E-cadherin-β-catenin association at the membrane [46], the role of N-terminally Ser/Thr phosphorylated β-catenin at the cell junctions remains an open question. Adherens junctions are dynamically modulated [44], [45] and while N-terminally phosphorylated β-catenin may not be necessary for stable cell-cell adhesions, it may be required for junction formation [17] or recycling [47]. Interestingly, Wnt5a was recently proposed to promote β-catenin/E-cadherin association via CK1α-mediated phosphorylation of β-catenin at Ser45 but not Ser33 [20] which supports a role for Ser45 phospho-β-catenin in cell adhesion and also raises the possibility that localised phosphorylation at individual sites may lead to distinct functional outcomes.

The striking cytoplasmic distribution of phospho-β-catenin in mitotic cells, particularly for P41/45-β-catenin, suggests a role in cell-cycle regulation. Total β-catenin levels are reported to increase in early S phase and are maximal at G2/M [48], but the increase in the phospho-β-catenin is much more dramatic than any increase in the total β-catenin levels. However, it has been shown that many proteins become highly phosphorylated during M phase [49], therefore it is not yet clear whether N-terminal phosphorylation of this population of β-catenin is functional or whether it is a bystander phosphorylation.

Our discovery that phospho-β-catenin accumulates with APC clusters at the ends of microtubules reveals an unexpected role for β-catenin in conjunction with microtubule-associated APC. APC localisation to the plus ends of growing microtubules is well established [33], [42] and is linked to its role in cell migration, polarization and microtubule stability [23]. While β-catenin and stabilised β-catenin mutants have been reported to be associated with peripheral APC clusters [33], [50], [51], [52], its role at this location is not entirely clear. Conflicting reports have suggested that β-catenin contributes to accumulation of APC at membrane clusters [52] or interferes with APC function [51]. In addition, it is not known whether the multiple complexes that APC participates in exist in concert or as distinct complexes. A recent report indicates that phospho-β-catenin is also localised in clusters at microtubule ends in endothelial cells and that CK1- and GSK3β-mediated phosphorylation of APC, but not β-catenin, appears to promote cell migration in a process that is independent of β-catenin-mediated transcription [24]. Our results support this notion except that in endothelial cells, APC clusters appeared to be linked to the proteasomal degradation of APC and β-catenin [24]. Since we have shown that extending pseudopodia contain APC, but not axin, it is clear that the APC at microtubule extensions is distinct from the axin-APC complex [38], [53] in epithelial cells. Unlike endothelial cells, APC is not present at the lateral membrane in MDCK cells. Furthermore, we have shown that phospho-β-catenin is localised to Ca^2+^-dependent cell contacts and forms a complex with E-cadherin whereas phospho-β-catenin was not detected in VE-cadherin complexes [24], underscoring the differences between the cell types which may explain the different conclusions.

We propose that the form of N-terminally phosphorylated β-catenin which accumulates with APC at cell extensions is not targeted for degradation but may regulate microtubule-associated APC function. Stabilised β-catenin has previously been implicated in inhibition of APC function in neurite formation and growth [51]. However, we did not detect altered APC at microtubule extensions upon inhibition of β-catenin phosphorylation. Our data suggest that unlike stabilised β-catenin, phospho-β-catenin does not impede APC function at microtubules.

Our experiments show that GSK3β phosphorylates the β-catenin that accumulates with APC at microtubule ends. The action of GSK3β is intriguing. GSK3β phosphorylation of APC is reported to abolish microtubule binding [54] and localised inactivation of GSK3β in migrating astrocytes and neurite extensions leads to accumulation of APC in cell polarisation and axon growth, respectively [39], [55]. In contrast, Wnt-induced GSK3β inactivation in neuronal growth cones, and CK1- and GSK3β-inhibition in endothelial cells, leads to decreased APC at microtubule plus ends [24], [56]. Different levels of GSK3β inhibition are proposed to exert different responses depending on cell context or cell type [14], [56]. We observed reduced phospho-β-catenin associated with APC in epithelial cells within 5 min of GSK3β inhibition, indicating that turnover of phosphorylation at these sites is rapid, and this was maintained during 6 h of treatment. We did not observe changes in APC clusters during this time frame. In contrast, previous reports identified accumulation of APC after 8 h to 24 h treatments [55], [57]. We also show prominent accumulation of phospho-β-catenin with APC at the leading edge of cells stimulated to migrate for 1–2 h indicating that GSK3β phosphorylates β-catenin associated with APC in migrating cells. While depletion of β-catenin did not result in differences in endothelial cell migration upon GSK3β inhibition [24], β-catenin has been strongly implicated in epithelial cell migration [58] and our current results link phospho-β-catenin to APC migration/adhesion processes that are independent of axin and β-catenin degradation. Thus GSK3β is important in regulating APC at microtubules, but is also involved in phosphorylation of β-catenin. As functional APC is required for localised phospho-β-catenin at cell protrusions, this process must also involve APC.

The rapid loss of phospho-β-catenin at the protrusion tips following GSK3β inhibition suggests the presence of a phosphatase, as proposed in an earlier study [17]. PP2A has been linked to β-catenin and the degradation complex [59], [60], [61], where APC has been proposed to protect phosphorylated β-catenin from PP2A dephosphorylation thus enabling it to be ubiquitinated and degraded [62]. Whether APC plays a similar role at microtubule plus ends remains to be determined, but we did detect significant levels of PP2A in isolated pseudopodia (MCF, unpublished) and inhibition of PP2A resulted in increased phospho-β-catenin staining in clusters coincident with APC. Identifying the molecular mechanisms that regulate localised phosphorylation of β-catenin at the microtubules will be an important challenge in the future.

While our data showing that axin is excluded from APC-rich protrusions suggests that phospho-β-catenin is not always targeted for proteasomal-mediated destruction, we cannot rule out a model whereby phosphorylation of β-catenin earmarks the protein for destruction by non-axin-mediated mechanisms. One such scaffold complex, containing presenilin, has previously been implicated in mediating phosphorylation of β-catenin [63]. However, while treatment with proteasome inhibitors increases the levels of phospho-β-catenin co-incident with axin puncta [38], clearly demonstrating regulation of these populations by proteasomal degradation, we observed no similar increase in the levels of the APC-rich protrusions-associated population of phospho-β-catenin due to treatment with proteasome inhibitors. Instead, the population associated with APC is prominent in actively migrating cells, implying that it has an active role in cell migration. Although surprising, our data also shows that kinases other than the CK1α/GSK3β combination can phosphorylate β-catenin, which may result in β-catenin destruction or mark distinct functions, such as in cell migration and/or adhesion processes. It should be noted that the rapid dephosphorylation of β-catenin in the presence of GSK3β inhibitors is also reversed rapidly when the inhibition is removed, suggesting that the β-catenin in the protrusions may not be destroyed.

Overall, our studies implicate phosphorylated β-catenin in cell adhesion and at the ends of APC-associated HGF- and wound healing-induced cell protrusions. The latter process requires functional APC and is perturbed in cancer cells containing mutated APC (Fig. 7). Regulation of localised phospho-β-catenin may contribute to the tumour suppressor activity of APC. Whether accumulation of APC and phospho-β-catenin at the leading edge is controlled by Wnt signalling will require further investigation. We propose that phosphorylation of β-catenin does not only lead to its degradation but provides a level of regulation for β-catenin function in distinct cellular processes. This premise is supported by our recent identification of the formation of a Wnt3a-induced phospho-β-catenin-APC-α-catenin complex that resides close to the plasma membrane and appears to be involved in cell-cell adhesion (Layton et al., submitted). The demonstration of localised phosphorylated forms of β-catenin provides important insight into understanding the complexities of β-catenin signalling in colon cancer.

## Materials and Methods

### Antibodies

The anti-phospho-β-catenin P41/45 and P33/37/41 antibodies were from Cell Signaling (9565 and 9561, respectively). The APC monoclonal antibody (APC-NT) was raised against amino acids 1–61 of human APC with an N-terminal Flag epitope tag, produced in bacteria (Elliott, Catimel and Faux, unpublished). Commercial antibodies were obtained from Santa Cruz (anti-APC (H290) and CK1α), Zymed (axin-1), BD Transduction Laboratories (β-catenin (19920/610153), GSK3β (G22320), E-cadherin, nucleoporin, PP2A), and Sigma (actin, clone AC-40 and β-tubulin, clone 2.1). AlexaFluor-conjugated secondary antibodies were from Molecular Probes and IR Dye-conjugated secondary antibodies were from LiCor Biosciences.

### Cell culture and treatments

MDCK cells [64] were grown in DMEM supplemented with 10% foetal calf serum (FCS) (DMEM+10%FCS) and 1% penicillin/streptomycin. Colorectal carcinoma cell lines SW480 [65], HCT116 and LIM cells: LIM2405 [66], LIM2537, LIM1215, LIM1899, LIM2551 [30] were grown in RPMI supplemented with 0.01 µg/ml thioglycerol, 0.025 U/ml insulin, 1 µg/ml hydrocortisone, 10% FCS and 1% penicillin/streptomycin.

For Ca^2+^ chelation experiments, MDCK cells were serum starved for 24 h and incubated with 4 mM EGTA, 1 mM MgCl_2_ in DMEM or control DMEM for 2 h. For GSK3β inhibitor experiments, cells were incubated with 2 nM Kenpaullone (Calbiochem) or dimethyl sulfoxide (DMSO) control, 30 mM LiCl or 30 mM NaCl, for the indicated times. For inhibition of CK1, cells were incubated with 100 µM D4476 (Calbiochem) diluted as described [67] and 5–10 µM IC261 (Calbiochem) or dimethyl sulfoxide (DMSO) control. For inhibitor washout experiments, MDCK cells were pretreated with 30 mM LiCl or NaCl for 30 min, cells were washed 2× and incubated with DMEM+10%FCS for 0, 5, 10 and 20 min. Scratch wound assays were performed by plating MDCK cells on optical glass coverslips. Confluent monolayers were scratched with a plastic tip, washed 2× DMEM and incubated with DMEM +10%FCS. The cells were fixed at 0, 1, 2.5 or 6 h. For HGF stimulation, MDCK cells were serum starved for 24 h and incubated with 2 ng/ml HGF (R and D Systems) for 6 h.

To deplete APC using RNA interference (RNAi), MDCK cells were transfected with small interfering RNA (siRNA) directed against APC with Lipofectamine 2000 (Invitrogen, Carlsbad, CA, USA) according to manufacturer's instructions using 0.3 µM siRNA targeting canine APC (Ambion) or 0.3 µM non-targeting siRNA. Predesigned Dharmacon siRNA pools targeting β-catenin (CTNNB1, L-003482-00-0005, NM_001904) and non-target control siRNA pools (D-001810-20) were used to deplete β-catenin in MDCK cells. Cells were harvested for immunoblot analysis or fixed for immunofluorescence 48 h after siRNA treatment. The sequences of siRNA oligonucleotides were: siAPC 1: 5′-GGAAUCAACCCUCAAAAGUtt-3′, siAPC 2: 5′-GCACACUGCACUGAGAAUAtt-3′, siAPC 3 5′-GCACACUGCACUGAGAAUAtt-3′ (Ambion).

### Confocal fluorescence microscopy

Cells were immunostained as described previously [68] with the indicated primary antibodies. AlexaFluor488-, 546-, 633- and 405-conjugated secondary antibodies were used for immunofluorescence analysis. For peptide competition experiments, P41/45 and P33/37/41 phospho-β-catenin antibodies were preincubated without and with P41/45 and P33/37/41 peptides (Cell Signalling), respectively, for 30 min. For immunostaining with antibodies raised in the same species, cells were first incubated with APC (APC-NT) mouse monoclonal antibodies for 1 hour, washed 3× 0.2%BSA/PBS, incubated with anti-mouse AlexaFluor-546 antibodies for 1 h, and then washed 4×0.2% BSA/PBS before incubating with β-tubulin or E-cadherin mouse monoclonal antibodies for 1 h. Cells were washed 3×0.2% BSA/PBS and incubated with anti-mouse AlexaFluor-405 antibodies for 1 h, washed 3×0.2% BSA/PBS. Cells were co-stained with DAPI or TO-PRO-3 Iodide (642/661 Molecular Probes) to visualise nuclei. Immunofluorescence staining was detected in successive focal planes using Nikon C1 or Olympus FV1000 confocal microscopes. Double- and triple- labelled images were detected using standard filter sets and laser lines. Cells were imaged with Nikon Plan Apo 60× (NA1.4), Nikon TIRF 100× (NA1.45), Olympus 60× (NA1.35) oil or Olympus 60× (NA1.35) water immersion lenses.

### Co-immunoprecipitation and immunoblot analysis

MDCK cells were lysed in ice-cold Lysis Buffer (20 mM HEPES, pH7.4 containing 150 mM NaCl, 5 mM EDTA, 1% Na deoxycholate, 1% Triton-X100, a phosphatase inhibitor cocktail (1 mM Na Vanadate, 10 mM NaF, 5 mM Na pyrophosphate, 5 mM β-glyercophosphate, 10 nM okadaic acid), and Complete (Roche) EDTA-free protease inhibitor cocktail). Lysates were clarified by microcentrifugation at 16,000 *g* for 30 min at 4°C. For immunoprecipitations, the lysates were incubated with the indicated antibodies (1–2 µg) for 16 h at 4°C and with protein A- or G-Sepharose (10% slurry) (Pharmacia) for the final 30 min 4°C. Precipitated proteins were eluted with 2× SDS-PAGE sample buffer and boiled for 10 minutes. Cell lysates and immunoprecipitated proteins were analysed by SDS-PAGE using 3–8% TrisAcetate or 4–12% Bis-Tris NuPAGE gels (Novex). Proteins were detected using the indicated primary antibodies and the Odyssey infrared imaging system (Odyssey). Protein bands were quantitated by densitometry, and analysed using GeneTools software.

### Purification of APC-rich protrusions

Pseudopodia were extracted as described by [37]. To monitor APC rich protrusions, serum starved MDCK cells (1×10^5^ or 1×10^6^) were placed in the upper compartment of a chamber (12 or 24 mm, respectively) containing a 3.0-µm porous polycarbonate membrane insert. Cells were allowed to attach for 2 h with 2 ng/ml HGF (R and D Systems), and then inserts placed in chambers containing serum free DMEM or DMEM+10%FCS. Cells were allowed to extend pseudopodia through the pores for 24 h. Pseudopodia (PS) and cell bodies (CB) on the lower- and upper-surface of the filter were immunostained using standard methods, after first removing upper and lower-surface proteins, respectively, as described [37]. Co-staining with DAPI revealed nuclei on the upper surface (cell body) but not the undersurface (pseudopodia).

To extract proteins, 5×10^5^ cells were induced to extend protrusions across the filter. Proteins on the lower-(PS) and upper-(CB) surface of the filter were scraped directly into Lysis Buffer after first removing upper- and lower-surface proteins, respectively, by wiping the surface as described [37]. We typically obtain 20 µg of protein from pseudopodia from each 24 mm well which represents ∼5% of total cellular protein, similar to protein amounts reported by [37].

## Supporting Information

Figure S1 Reduction of phospho-β-catenin following β-catenin siRNA treatment MDCK cells were treated with siRNA directed against β-catenin or a control sequence. Cells were harvested for immunoblot analysis 72 h after siRNA transfection. Shown is β-catenin immunoblot analysis of 1%Triton, 1% Deoxycholate extracts from control and β-catenin siRNA treated MDCK cells. In β-catenin siRNA treated cells, β-catenin was decreased ∼70% (n = 3).
(6.53 MB TIF)

Figure S2 Phospho-β-catenin antibodies are specific. (A) P41/45 β-catenin antibody staining demonstrates cell membrane localisation in the absence but not in the presence of the competing phospho-peptide. MDCK cells were fixed and immunostained with P41/45-β-catenin antibodies preincubated with P41/45-β-catenin blocking peptide. Bottom panels show mitotic cells co-stained with DAPI showing the specific increase in cytoplasmic phospho-β-catenin in mitotic cells. Upper panels are confocal Z projections, lower panels are single confocal sections of immunostained MDCK cells, scale bar 20 µm. (B) P33/37/41 β-catenin antibody staining demonstrates cell membrane (arrow), centrosome (arrowhead), intracellular cluster (dashed arrow) as well as mid-body bridge (not shown), in the absence but not in the presence of the competing phosphopeptide. MDCK cells were fixed and immunostained with P33/37/41 β-catenin antibdies preincubated with P33/37/41 β-catenin blocking peptide. Shown are confocal Z projections of immunostained cells, scale bar 20 µm. Phospho-peptides contain the same sequence as the antigen used to raise phospho-β-catenin antibodies.
(3.77 MB TIF)

Figure S3 Phospho-β-catenin antibodies recognise phosphorylated but not unphosphorylated β-catenin. (A) Immunoblot analysis of purified recombinant wild type β-catenin and S45A -β-catenin phosphorylated with CK1α alone, GSK3β alone and CK1α+GSK3β probed with β-catenin and phospho-β-catenin antibodies, as indicated. P41/45 phospho-β-catenin antibodies recognise β-catenin phosphorylated by CK1α alone and CK1α+GSK3β but not GSKβ alone; P33/37/41 phospho-β-catenin antibodies recognise β-catenin phosphorylated by CK1α+GSK3β but not CK1α or GSK3β alone. S45A β-catenin is not phosphorylated by CK1α and is therefore not recognised by P41/45 or P33/37/41-β-catenin antibodies. Full length mouse β-catenin (residues 1–782, accession number Q02248), subcloned into pET-DUET as an Asc1/Not1 fragment, was expressed in E.coli BL21 (DE3) cells as an N-terminal His6 fusion protein was purified using nickel affinity and size exclusion chromatography. Active CK1α (a kind gift from Dr Jorge Allende) was expressed in E.coli BL21 (DE3) and part-purified as a His6 fusion protein by Ni-affinity chromatography. GSK3β with an N-terminal Flag epitope tag was subcloned into the baculovirus vector pBlueBac4 (Invitrogen) and recombinant protein produced in Sf9 cells. Flag-GSK3β was purified by affinity chromatography using anti-Flag-M2 agarose (Sigma) followed by size exclusion chromatography. 20 pmols purified recombinant His6-β-catenin or His6-β-catenin S45A mutant was phosphorylated in the presence of 1 mM ATP, 4 mM MgCl2, 150 mM NaCl, 20 mM Tris-HCl, pH 7.5 using recombinant His6-CK1α and Flag-GSK3β. Reactions were performed in a final volume of 20 µl at room temperature for 30 min, terminated by the addition of SDS-PAGE loading buffer, and separated by SDS-PAGE (4–12% Bis-Tris). Phosphate incorporation was verified by performing the reaction in the presence of 2 −μCi γ^32^P-ATP followed by phospho-imaging, or by immunoblot with the indicated antibodies. (B) Autoradiograph of phosphorylated β-catenin from (A) showing incorporation of 32P phosphate followingphosphorylation by CK1α and CK1α+GSK3β but not GSK3β alone.
(1.68 MB TIF)

Figure S4 Specificity of phospho-β-catenin antibodies (A) LIM2551 cells, containing mutated β-catenin that results in deletion of amino acids 5–72 [30], show little to no detectable P41/45 β-catenin and P33/37/41 β-catenin in interphase cells. Shown are confocal sections of LIM2551 cells immunostained with P41/45 β-catenin (top) and P33/37/41 β-catenin (bottom) antibodies and costained with DAPI, scale bar 20 µm. (B) P33/37/41 β-catenin antibodies stain centrosomes and mid-body bridges in mitotic LIM2551 cells. It is important to note that as β-catenin does not contain the P33/37/41 β-catenin epitope, this staining is not related to β-catenin. Shown are confocal micrographs of LIM2551 cells immunostained with P33/37/41 β-catenin and β-tubulin antibodies and co-stained with DAPI to visualise DNA, scale bar 5 µm.
(1.47 MB TIF)

Figure S5 Detection of phosphorylated-β-catenin in colon cancer cells. Immunoblots of MDCK and colon cancer cell lysates demonstrate that the major band detected by P41/45- and P33/37/41-β-catenin antibodies co-migrates with β-catenin. Filters were probed with antibodies to the indicated proteins. Cells containing mutations in APC (LIM2537 and SW480) have detectable phospho-β-catenin. Cells containing β-catenin mutations at S45 (LIM1899 and HCT116) do not have detectable P41/45-β-catenin but are still phosphorylated at P33/37/41. LIM1215 cells contain a mutation at T41. Note that in LIM2551 cells, β-cateninΔ5–72 migrates faster by SDS-PAGE consistent with the size of the mutated protein and is not detected by P41/45 or P33/37/41. Cells treated with 30 mM LiCl do not show any change in P41/45-β-catenin, as expected, but show reduced P33/37/41-β-catenin as detected by P41/45- and P33/37/41-β-catenin antibodies, respectively. Shown are representatives of at least three separate experiments.
(1.98 MB TIF)

Figure S6 Axin is localised to cytoplasmic puncta and not at cell junctions or the ends of cell extensions in MDCK cells. MDCK cells were serum starved and untreated (control (A and C)) or treated with HGF (2 ng/ml) for 6 h (B and D), fixed and immunostained with axin and β-tubulin (A and B) and axin or APC (C and D) antibodies and co-stained with DAPI to visualise nuclei. Left panels are grey scale images of axin; Right panels are merged images, axin (green), β-tubulin/APC (red), nuclei (blue). Axin was not detected at the cell periphery or at the ends of microtubules in control or HGF stimulated cells. Scale bars 20 µm.
(6.25 MB TIF)

Figure S7 Phosphorylated β-catenin is localised in clusters to the ends of microtubules and is coincident with APC. MDCK cells were immunostained with antibodies to P41/45- (A) or P33/37/41-β-catenin (B) (green), APC (magenta) and β-tubulin (blue) and in (B) co-stained with TO-PRO-3 iodide to visualise nuclei (cyan). APC clusters and coincident phospho-β-catenin (white) are indicated with green arrowheads. Note that APC decorates the ends of microtubules; in tight colonies, the cell protrusions are not distinct and extend across the epithelial monolayer so it is sometimes not obvious that the APC clusters are at the ends of microtubules even when cells are costained with tubulin (see A). However, at the edges of colonies, the ends of microtubules are distinct and the APC clusters at cell protrusions are clearly visible.Shown are confocal sections, scale bar 20 µm.
(8.56 MB TIF)

Figure S8 Inhibition of β-catenin phosphorylation in MDCK cells. Lysates from MDCK cells treated with GSK3β and CK1α inhibitors and immunoblotted as indicated. (A) P33/37/41-β-catenin is reduced following treatment with all kinase inhibitors, but most strongly following inhibition with GSK3β inhibitors, LiCl and kenpaullone. (B) P41/45-β-catenin is reduced following treatment with D4476, and reduced slightly with IC261, but not by treatment with GSK3β inhibitors, LiCl and kenpaullone. C) P41/45-β-catenin remains localised in sub-populations at cell junctions (arrows) and in clusters coincident with APC (arrowheads) following treatment with GSK3β inhibitor, LiCl. Scale 10 µm.
(3.21 MB TIF)

Figure S9 Inhibition of CK1 results in loss of phospho-β-catenin at cell-cell contacts. MDCK cells were treated with vehicle control or D4476 (100 µM) for 1 h, fixed and immunostained with antibodies to P41/45 β-catenin or P33/37/41β-catenin (green), APC or E-cadherin (red), and co-stained with DAPI to visualise nuclei. (A) P41/45 β-catenin (green), APC (red), nuclei (blue). (B) P41/45 β-catenin (green), E-cadherin (red), nuclei (blue). (C) P33/37/41 β-catenin (green), APC (red), nuclei (blue). Phospho β-catenin cell contact staining, indicated by solid arrows, is lost in D4476 treated cells but co-incident APC staining, indicated by dashed arrows, and cytoplasmic staining in mitotic cells (*) remains after treatment. Shown are confocal sections, scale bars 20 µm.
(7.60 MB TIF)

Figure S10 Inhibition of protein phosphatases results in an increase in P33/37/41-β-catenin associated with APC. (A) MDCK cells were pre-treated with 30 mM NaCl or LiCl for 30 min, cells were washed 2× and incubated in DME+10% FCS containing vehicle control or okadaic acid (20 nM). Cells were harvested at T0, 5, 10 and 20 min and lysates immunoblotted with P33/37/41-anti rabbit-IR800 and reprobed with actin-anti-mouse-IR700 antibodies. The numbers below each lane reflect normalised densitometry values. Shown is a representative of at least 3 independent experiments. There is a modest increase in phosphorylated (P33/37/41)-β-catenin in the presence of the phosphatase inhibitor. (B) MDCK cells were pre-treated with 30 mM NaCl or LiCl for 30 min and cells were washed 2× and either fixed (T0) or incubated in DME+10%FCS without and with Calyculin (20 nM) for 20 min. Fixed cells were immunostained with P33/37/41-β-catenin (green) and APC (red) antibodies and costained with DAPI. P33/37/41-β-catenin coincident with APC clusters at cell protrusions is reduced with LiCl treament and is partially restored after LiCl is washed out. Incubation with phosphatase inhibitor, Calyculin, results in increased P33/37/41-β-catenin in clusters coincident with APC. Representative fields for each treatment are shown in left panels. Arrowheads indicate APC clusters. Scale bar 20 µm. Cells were scored for coincident P33/37/41-β-catenin and APC staining and plotted in right panel. Shown is average +/− SD from three independent experiments, >100 cells per treatment group. **P<0.02, ***P<0.005, ****P = 0.00005 by Students t test.
(3.08 MB TIF)

Figure S11 APC is required for phospho-β-catenin localisation to the ends of microtubules. MDCK cells transfected with control and APC siRNA were immunostained for P33/37/41 β-catenin (green), APC (magenta) and E-cadherin (blue) with grey scale images shown below and enlarged in insets. Arrowheads indicate clusters of phospho-β-catenin at cell extensions coincident with APC in control but not APC depleted cells. White arrows indicate intact junctional phospho-β-catenin; green arrows indicate concentrations of phospho-β-catenin coincident with E-cadherin. Scale bars 20 µ (insets, 10 µ).
(5.37 MB TIF)
