# Supplementary figures and images for: Independent Interactions of Phosphorylated β-Catenin with E-Cadherin at Cell-Cell Contacts and APC at Cell Protrusions
Source: PLoS One. 2010 Nov 30;5(11):e14127. doi: 10.1371/journal.pone.0014127 (PMC2994709; doi:10.1371/journal.pone.0014127)

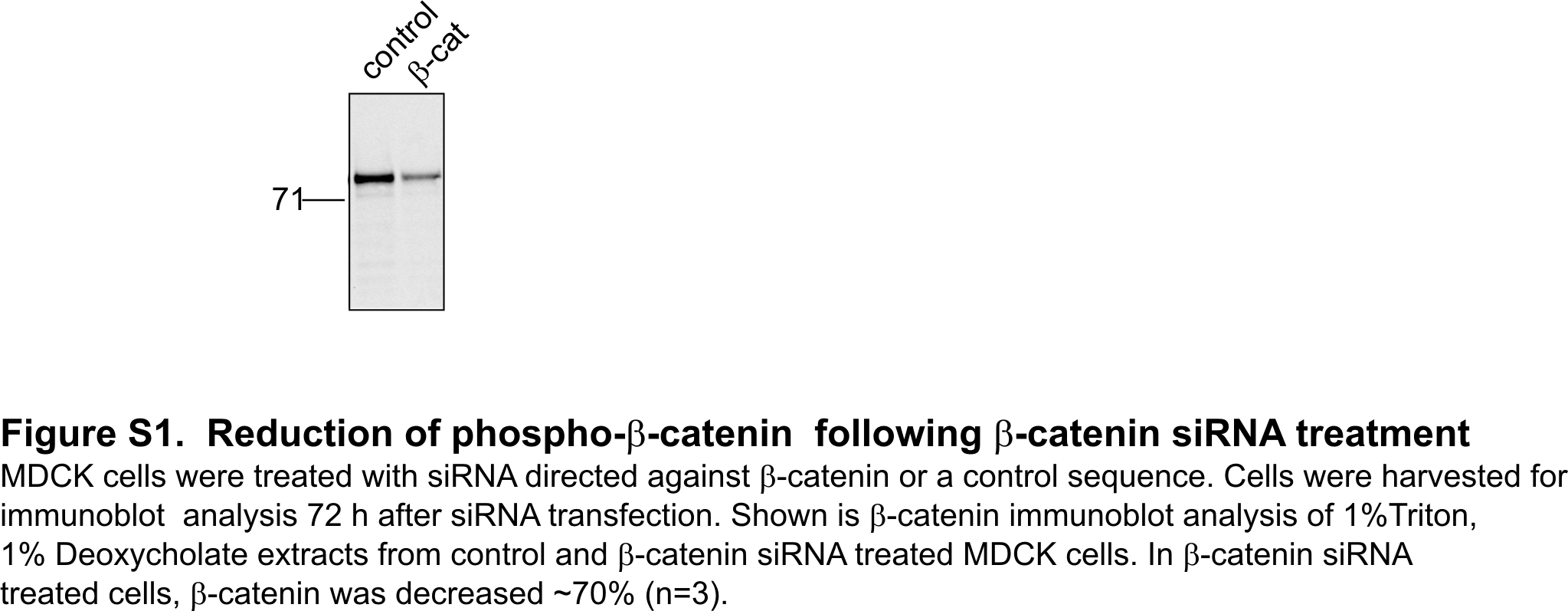

Supplement: Figure S1 — Reduction of phospho-β-catenin following β-catenin siRNA treatment MDCK cells were treated with siRNA directed against β-catenin or a control sequence. Cells were harvested for immunoblot analysis 72 h after siRNA transfection. Shown is β-catenin immunoblot analysis of 1%Triton, 1% Deoxycholate extracts from control and β-catenin siRNA treated MDCK cells. In β-catenin siRNA treated cells, β-catenin was decreased ∼70% (n = 3). (6.53 MB TIF) [file pone.0014127.s001.tif]

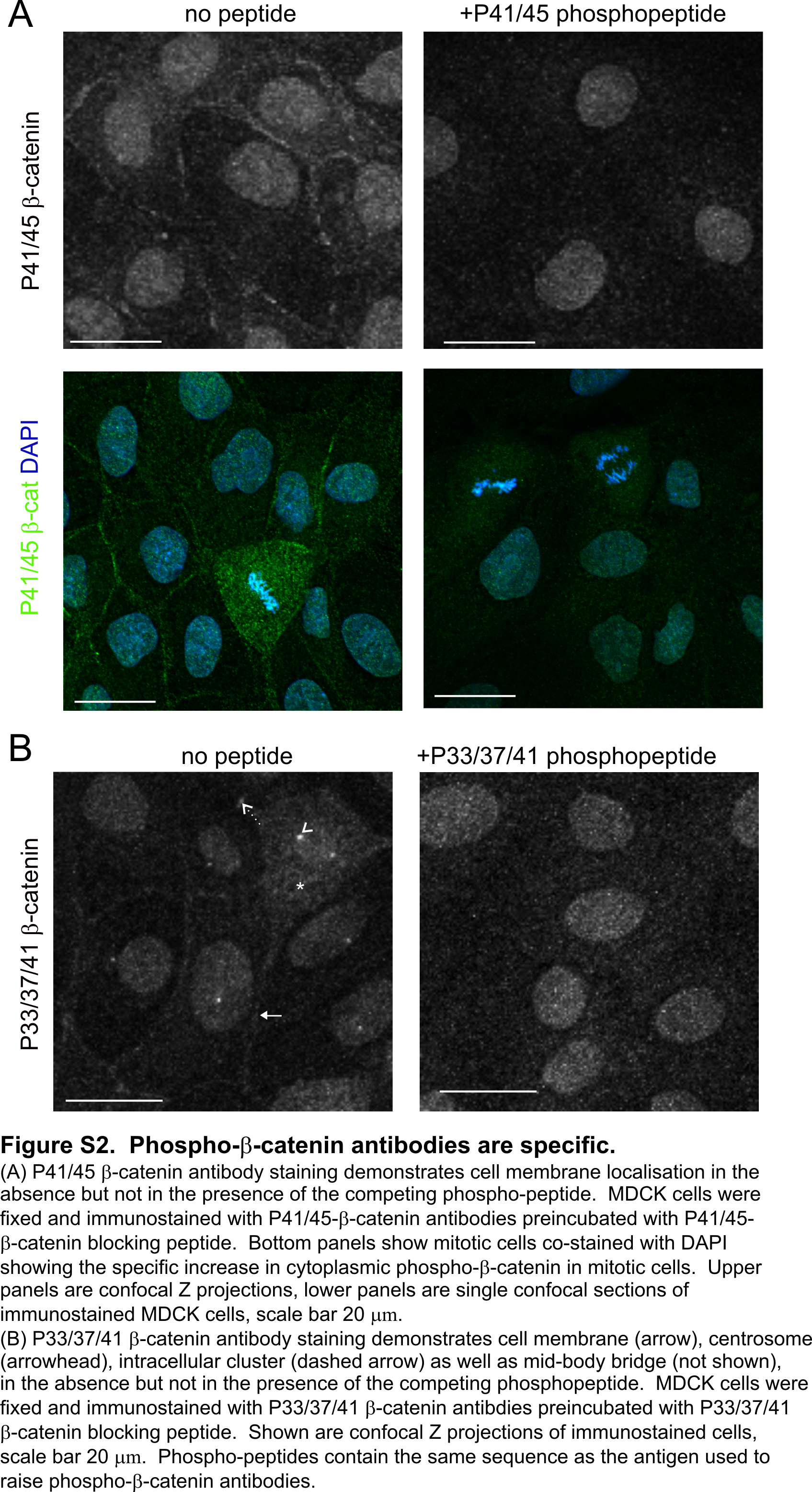

Supplement: Figure S2 — Phospho-β-catenin antibodies are specific. (A) P41/45 β-catenin antibody staining demonstrates cell membrane localisation in the absence but not in the presence of the competing phospho-peptide. MDCK cells were fixed and immunostained with P41/45-β-catenin antibodies preincubated with P41/45-β-catenin blocking peptide. Bottom panels show mitotic cells co-stained with DAPI showing the specific increase in cytoplasmic phospho-β-catenin in mitotic cells. Upper panels are confocal Z projections, lower panels are single confocal sections of immunostained MDCK cells, scale bar 20 µm. (B) P33/37/41 β-catenin antibody staining demonstrates cell membrane (arrow), centrosome (arrowhead), intracellular cluster (dashed arrow) as well as mid-body bridge (not shown), in the absence but not in the presence of the competing phosphopeptide. MDCK cells were fixed and immunostained with P33/37/41 β-catenin antibdies preincubated with P33/37/41 β-catenin blocking peptide. Shown are confocal Z projections of immunostained cells, scale bar 20 µm. Phospho-peptides contain the same sequence as the antigen used to raise phospho-β-catenin antibodies. (3.77 MB TIF) [file pone.0014127.s002.tif]

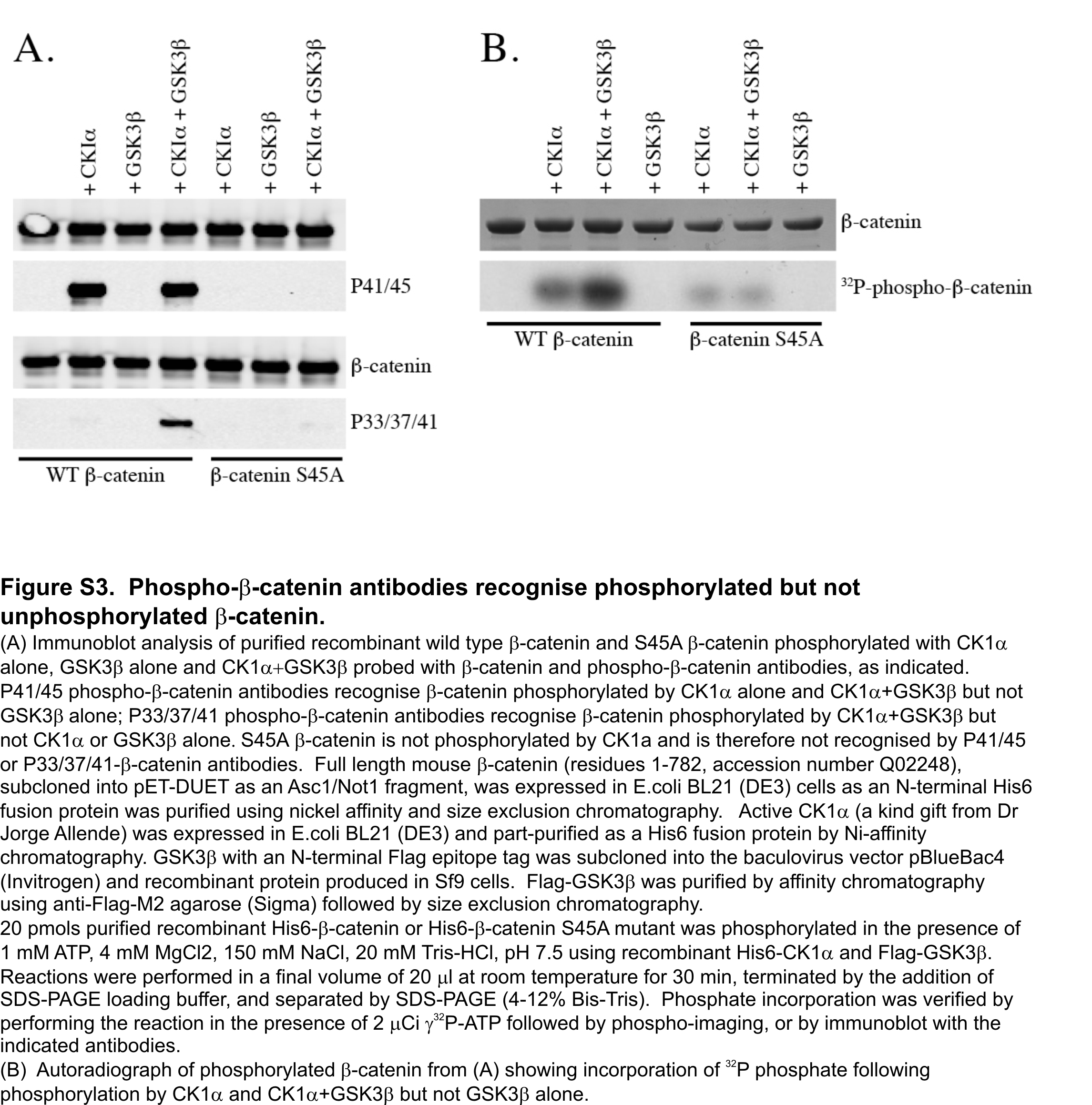

Supplement: Figure S3 — Phospho-β-catenin antibodies recognise phosphorylated but not unphosphorylated β-catenin. (A) Immunoblot analysis of purified recombinant wild type β-catenin and S45A -β-catenin phosphorylated with CK1α alone, GSK3β alone and CK1α+GSK3β probed with β-catenin and phospho-β-catenin antibodies, as indicated. P41/45 phospho-β-catenin antibodies recognise β-catenin phosphorylated by CK1α alone and CK1α+GSK3β but not GSKβ alone; P33/37/41 phospho-β-catenin antibodies recognise β-catenin phosphorylated by CK1α+GSK3β but not CK1α or GSK3β alone. S45A β-catenin is not phosphorylated by CK1α and is therefore not recognised by P41/45 or P33/37/41-β-catenin antibodies. Full length mouse β-catenin (residues 1–782, accession number Q02248), subcloned into pET-DUET as an Asc1/Not1 fragment, was expressed in E.coli BL21 (DE3) cells as an N-terminal His6 fusion protein was purified using nickel affinity and size exclusion chromatography. Active CK1α (a kind gift from Dr Jorge Allende) was expressed in E.coli BL21 (DE3) and part-purified as a His6 fusion protein by Ni-affinity chromatography. GSK3β with an N-terminal Flag epitope tag was subcloned into the baculovirus vector pBlueBac4 (Invitrogen) and recombinant protein produced in Sf9 cells. Flag-GSK3β was purified by affinity chromatography using anti-Flag-M2 agarose (Sigma) followed by size exclusion chromatography. 20 pmols purified recombinant His6-β-catenin or His6-β-catenin S45A mutant was phosphorylated in the presence of 1 mM ATP, 4 mM MgCl2, 150 mM NaCl, 20 mM Tris-HCl, pH 7.5 using recombinant His6-CK1α and Flag-GSK3β. Reactions were performed in a final volume of 20 µl at room temperature for 30 min, terminated by the addition of SDS-PAGE loading buffer, and separated by SDS-PAGE (4–12% Bis-Tris). Phosphate incorporation was verified by performing the reaction in the presence of 2 −μCi γ32P-ATP followed by phospho-imaging, or by immunoblot with the indicated antibodies. (B) Autoradiograph of phosphorylated β-catenin from [file pone.0014127.s003.tif]

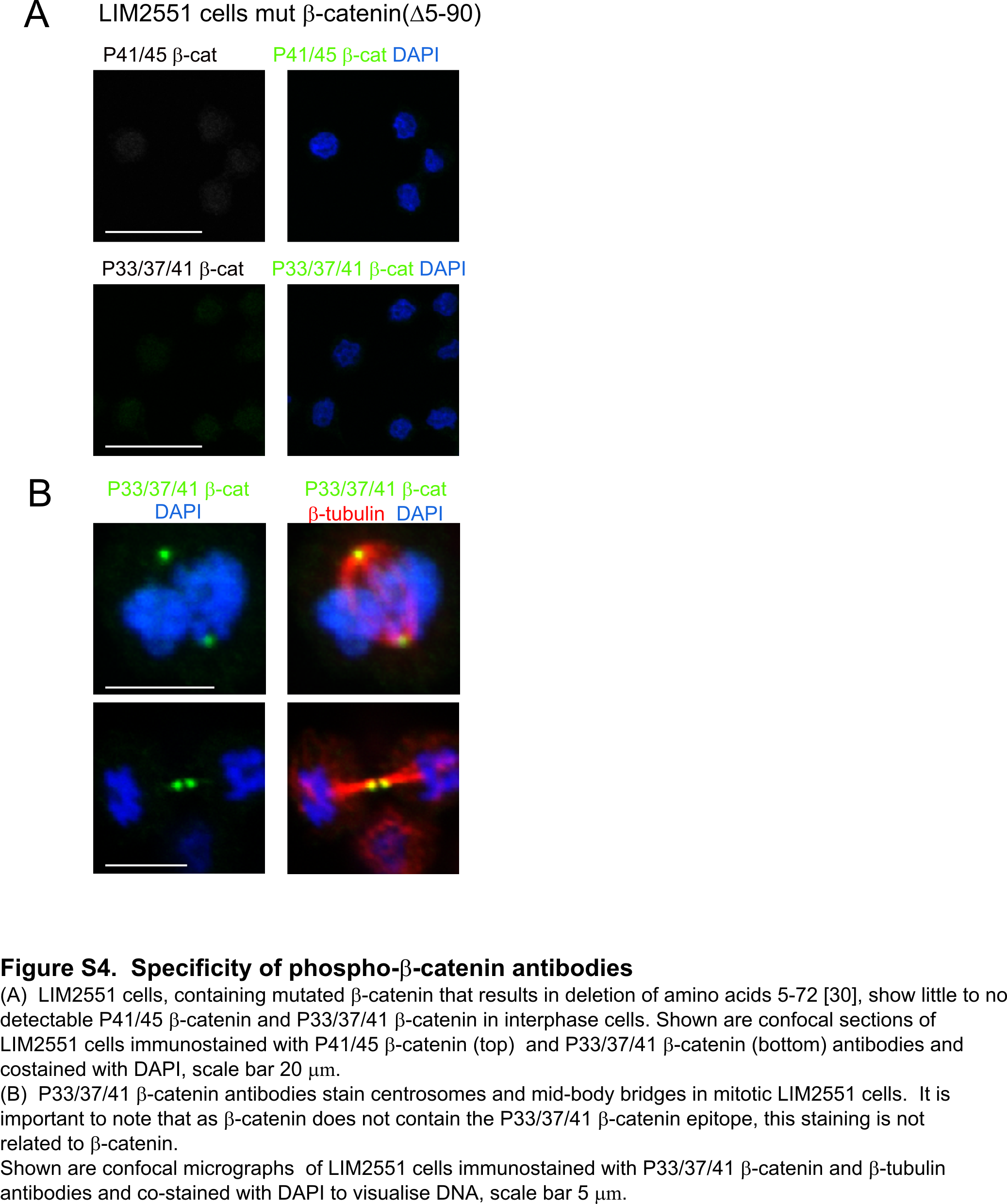

Supplement: Figure S4 — Specificity of phospho-β-catenin antibodies (A) LIM2551 cells, containing mutated β-catenin that results in deletion of amino acids 5–72 [30], show little to no detectable P41/45 β-catenin and P33/37/41 β-catenin in interphase cells. Shown are confocal sections of LIM2551 cells immunostained with P41/45 β-catenin (top) and P33/37/41 β-catenin (bottom) antibodies and costained with DAPI, scale bar 20 µm. (B) P33/37/41 β-catenin antibodies stain centrosomes and mid-body bridges in mitotic LIM2551 cells. It is important to note that as β-catenin does not contain the P33/37/41 β-catenin epitope, this staining is not related to β-catenin. Shown are confocal micrographs of LIM2551 cells immunostained with P33/37/41 β-catenin and β-tubulin antibodies and co-stained with DAPI to visualise DNA, scale bar 5 µm. (1.47 MB TIF) [file pone.0014127.s004.tif]

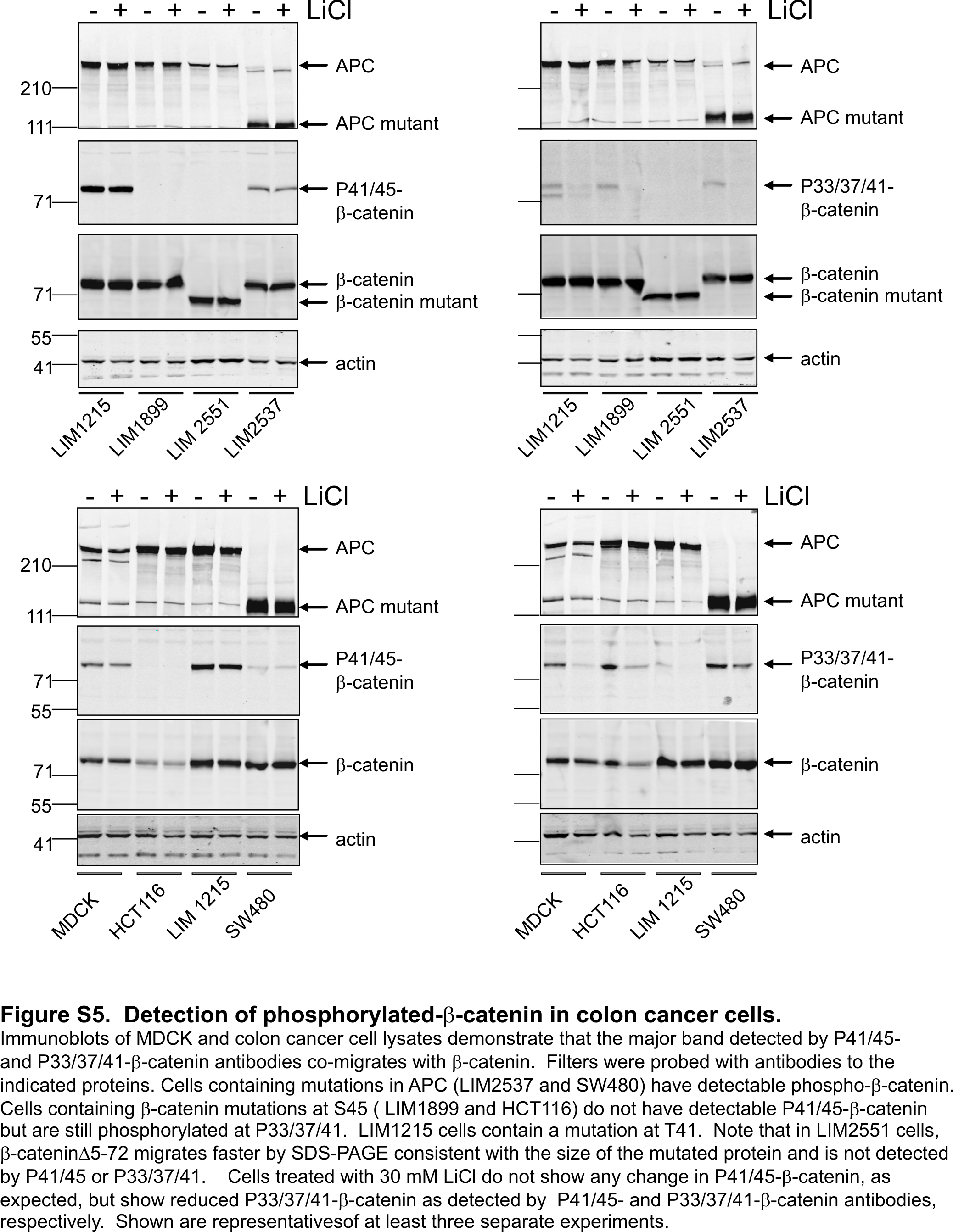

Supplement: Figure S5 — Detection of phosphorylated-β-catenin in colon cancer cells. Immunoblots of MDCK and colon cancer cell lysates demonstrate that the major band detected by P41/45- and P33/37/41-β-catenin antibodies co-migrates with β-catenin. Filters were probed with antibodies to the indicated proteins. Cells containing mutations in APC (LIM2537 and SW480) have detectable phospho-β-catenin. Cells containing β-catenin mutations at S45 (LIM1899 and HCT116) do not have detectable P41/45-β-catenin but are still phosphorylated at P33/37/41. LIM1215 cells contain a mutation at T41. Note that in LIM2551 cells, β-cateninΔ5–72 migrates faster by SDS-PAGE consistent with the size of the mutated protein and is not detected by P41/45 or P33/37/41. Cells treated with 30 mM LiCl do not show any change in P41/45-β-catenin, as expected, but show reduced P33/37/41-β-catenin as detected by P41/45- and P33/37/41-β-catenin antibodies, respectively. Shown are representatives of at least three separate experiments. (1.98 MB TIF) [file pone.0014127.s005.tif]

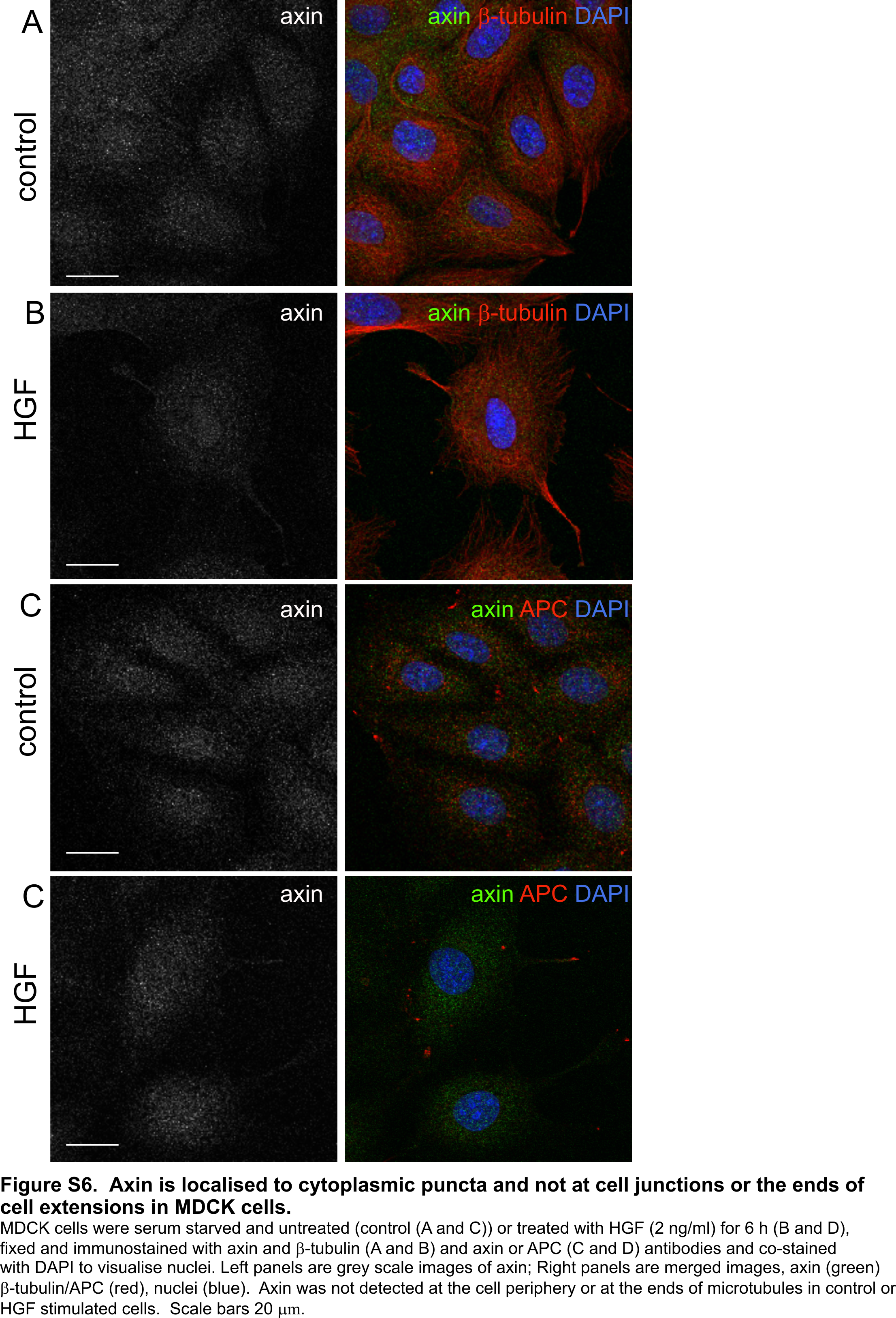

Supplement: Figure S6 — Axin is localised to cytoplasmic puncta and not at cell junctions or the ends of cell extensions in MDCK cells. MDCK cells were serum starved and untreated (control (A and C)) or treated with HGF (2 ng/ml) for 6 h (B and D), fixed and immunostained with axin and β-tubulin (A and B) and axin or APC (C and D) antibodies and co-stained with DAPI to visualise nuclei. Left panels are grey scale images of axin; Right panels are merged images, axin (green), β-tubulin/APC (red), nuclei (blue). Axin was not detected at the cell periphery or at the ends of microtubules in control or HGF stimulated cells. Scale bars 20 µm. (6.25 MB TIF) [file pone.0014127.s006.tif]

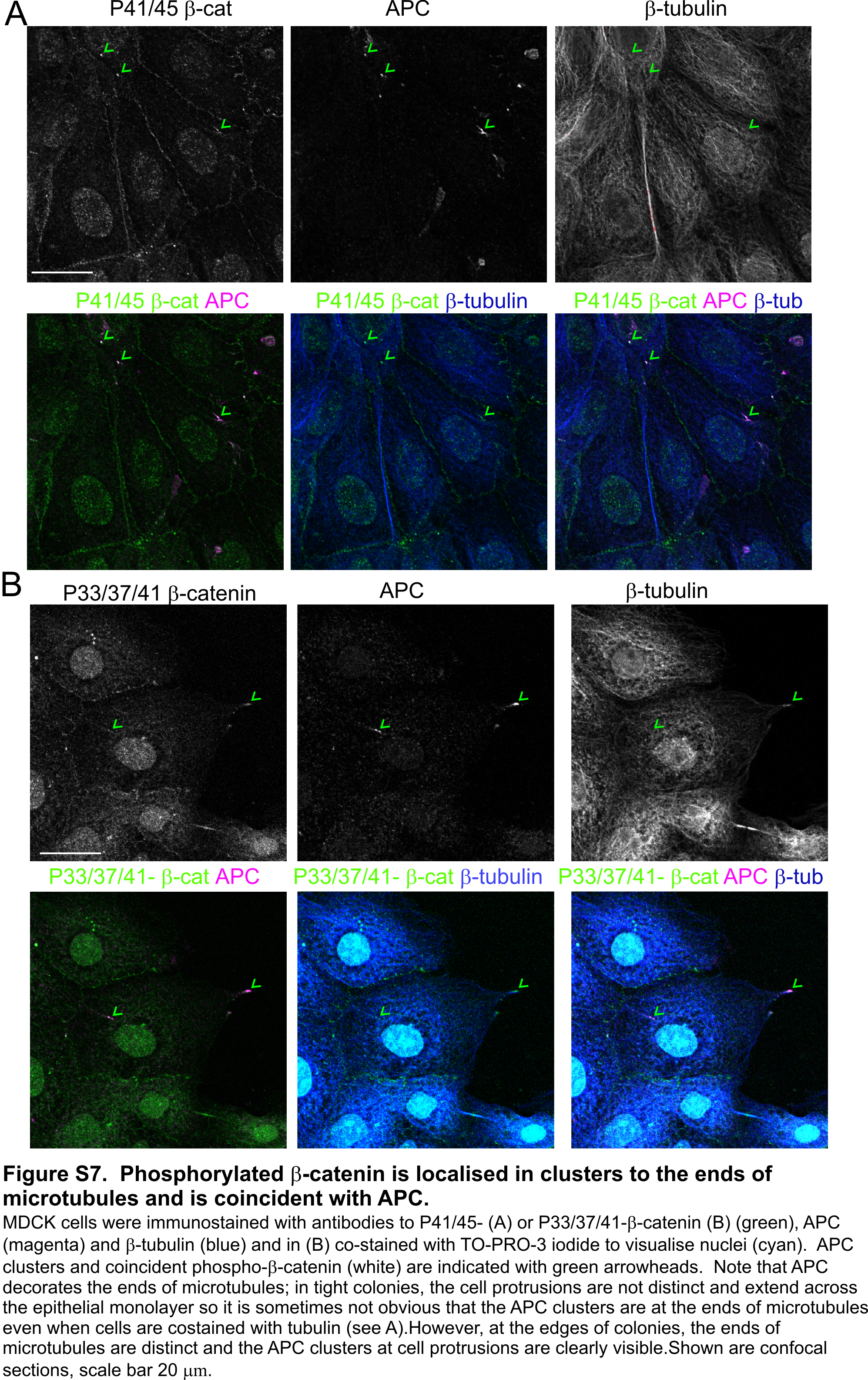

Supplement: Figure S7 — Phosphorylated β-catenin is localised in clusters to the ends of microtubules and is coincident with APC. MDCK cells were immunostained with antibodies to P41/45- (A) or P33/37/41-β-catenin (B) (green), APC (magenta) and β-tubulin (blue) and in (B) co-stained with TO-PRO-3 iodide to visualise nuclei (cyan). APC clusters and coincident phospho-β-catenin (white) are indicated with green arrowheads. Note that APC decorates the ends of microtubules; in tight colonies, the cell protrusions are not distinct and extend across the epithelial monolayer so it is sometimes not obvious that the APC clusters are at the ends of microtubules even when cells are costained with tubulin (see A). However, at the edges of colonies, the ends of microtubules are distinct and the APC clusters at cell protrusions are clearly visible.Shown are confocal sections, scale bar 20 µm. (8.56 MB TIF) [file pone.0014127.s007.tif]

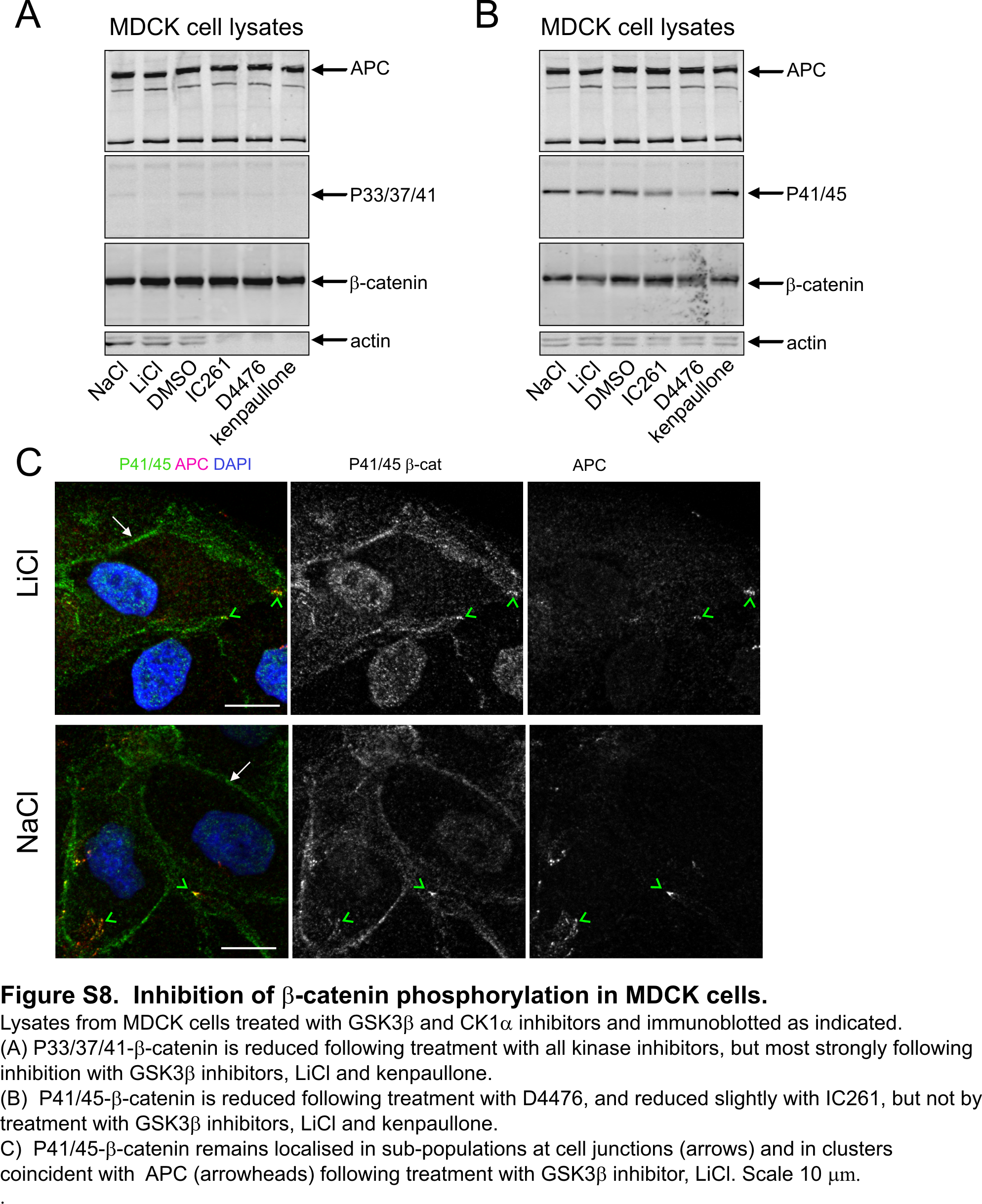

Supplement: Figure S8 — Inhibition of β-catenin phosphorylation in MDCK cells. Lysates from MDCK cells treated with GSK3β and CK1α inhibitors and immunoblotted as indicated. (A) P33/37/41-β-catenin is reduced following treatment with all kinase inhibitors, but most strongly following inhibition with GSK3β inhibitors, LiCl and kenpaullone. (B) P41/45-β-catenin is reduced following treatment with D4476, and reduced slightly with IC261, but not by treatment with GSK3β inhibitors, LiCl and kenpaullone. C) P41/45-β-catenin remains localised in sub-populations at cell junctions (arrows) and in clusters coincident with APC (arrowheads) following treatment with GSK3β inhibitor, LiCl. Scale 10 µm. (3.21 MB TIF) [file pone.0014127.s008.tif]

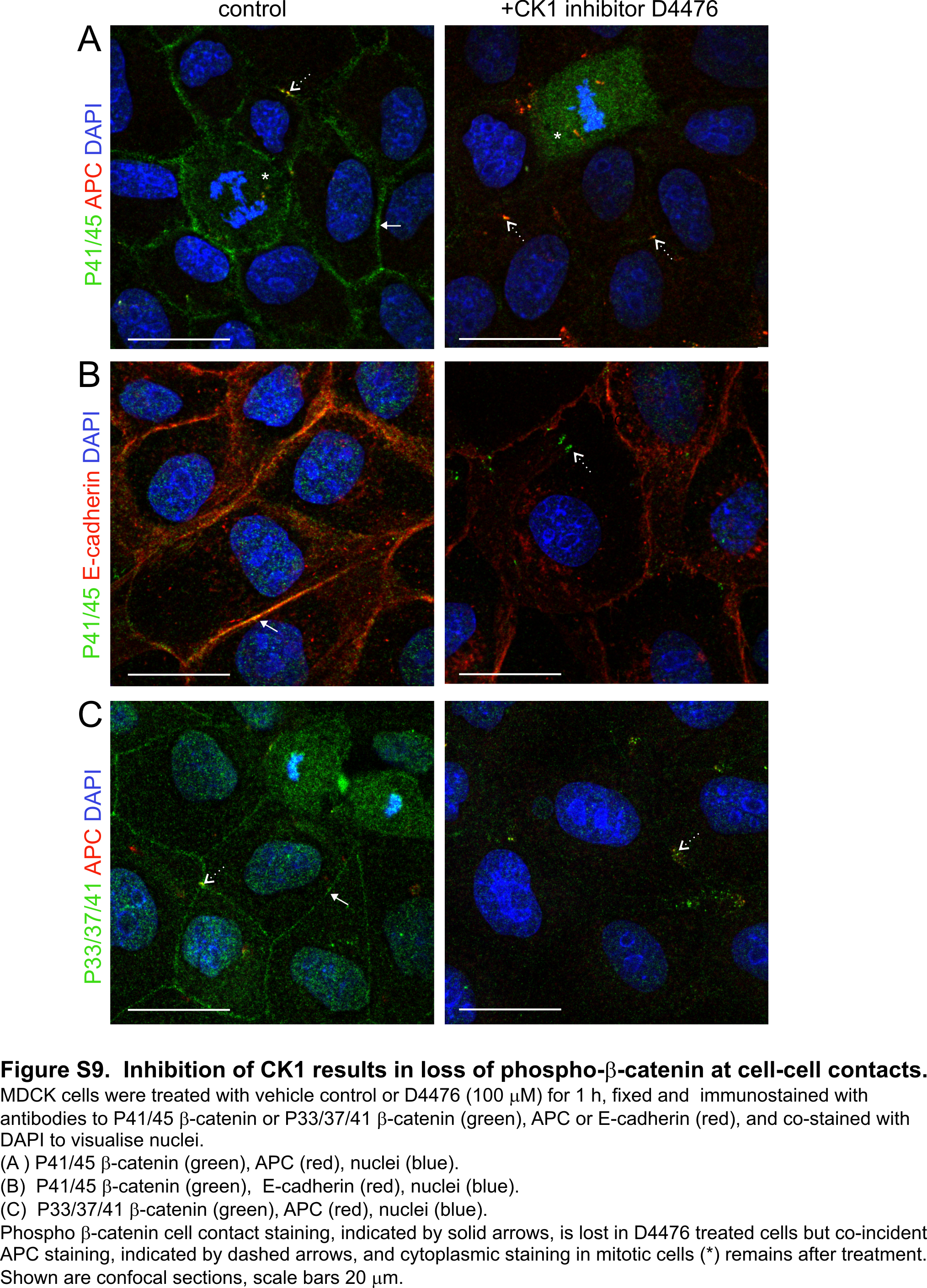

Supplement: Figure S9 — Inhibition of CK1 results in loss of phospho-β-catenin at cell-cell contacts. MDCK cells were treated with vehicle control or D4476 (100 µM) for 1 h, fixed and immunostained with antibodies to P41/45 β-catenin or P33/37/41β-catenin (green), APC or E-cadherin (red), and co-stained with DAPI to visualise nuclei. (A) P41/45 β-catenin (green), APC (red), nuclei (blue). (B) P41/45 β-catenin (green), E-cadherin (red), nuclei (blue). (C) P33/37/41 β-catenin (green), APC (red), nuclei (blue). Phospho β-catenin cell contact staining, indicated by solid arrows, is lost in D4476 treated cells but co-incident APC staining, indicated by dashed arrows, and cytoplasmic staining in mitotic cells (*) remains after treatment. Shown are confocal sections, scale bars 20 µm. (7.60 MB TIF) [file pone.0014127.s009.tif]

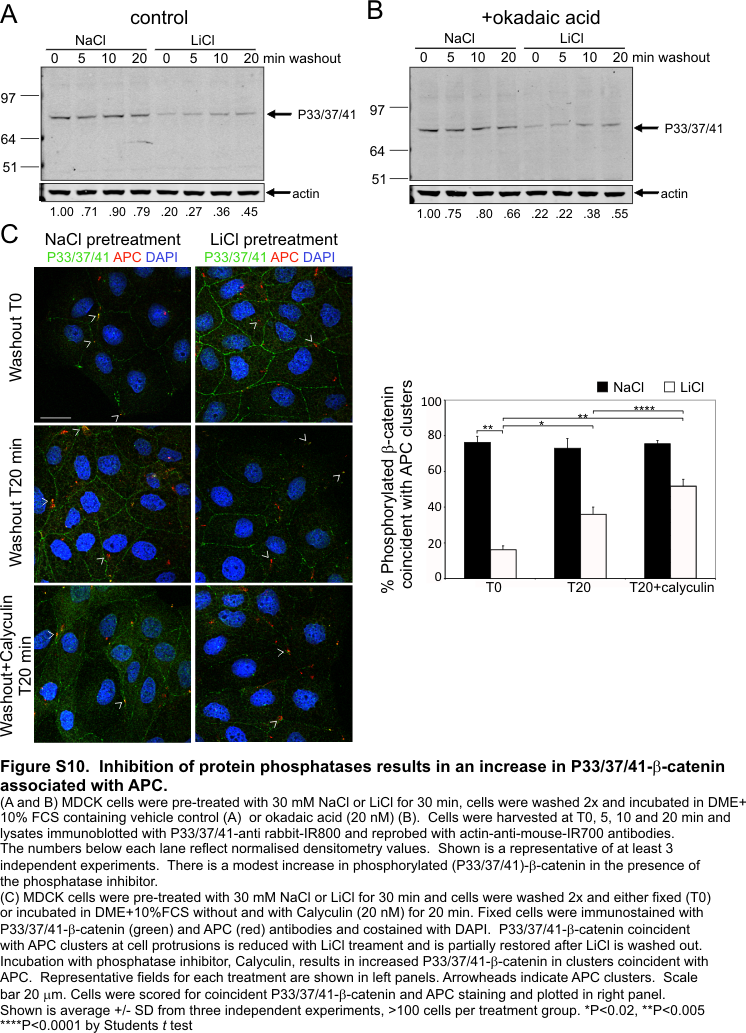

Supplement: Figure S10 — Inhibition of protein phosphatases results in an increase in P33/37/41-β-catenin associated with APC. (A) MDCK cells were pre-treated with 30 mM NaCl or LiCl for 30 min, cells were washed 2× and incubated in DME+10% FCS containing vehicle control or okadaic acid (20 nM). Cells were harvested at T0, 5, 10 and 20 min and lysates immunoblotted with P33/37/41-anti rabbit-IR800 and reprobed with actin-anti-mouse-IR700 antibodies. The numbers below each lane reflect normalised densitometry values. Shown is a representative of at least 3 independent experiments. There is a modest increase in phosphorylated (P33/37/41)-β-catenin in the presence of the phosphatase inhibitor. (B) MDCK cells were pre-treated with 30 mM NaCl or LiCl for 30 min and cells were washed 2× and either fixed (T0) or incubated in DME+10%FCS without and with Calyculin (20 nM) for 20 min. Fixed cells were immunostained with P33/37/41-β-catenin (green) and APC (red) antibodies and costained with DAPI. P33/37/41-β-catenin coincident with APC clusters at cell protrusions is reduced with LiCl treament and is partially restored after LiCl is washed out. Incubation with phosphatase inhibitor, Calyculin, results in increased P33/37/41-β-catenin in clusters coincident with APC. Representative fields for each treatment are shown in left panels. Arrowheads indicate APC clusters. Scale bar 20 µm. Cells were scored for coincident P33/37/41-β-catenin and APC staining and plotted in right panel. Shown is average +/− SD from three independent experiments, >100 cells per treatment group. **P<0.02, ***P<0.005, ****P = 0.00005 by Students t test. (3.08 MB TIF) [file pone.0014127.s010.tif]

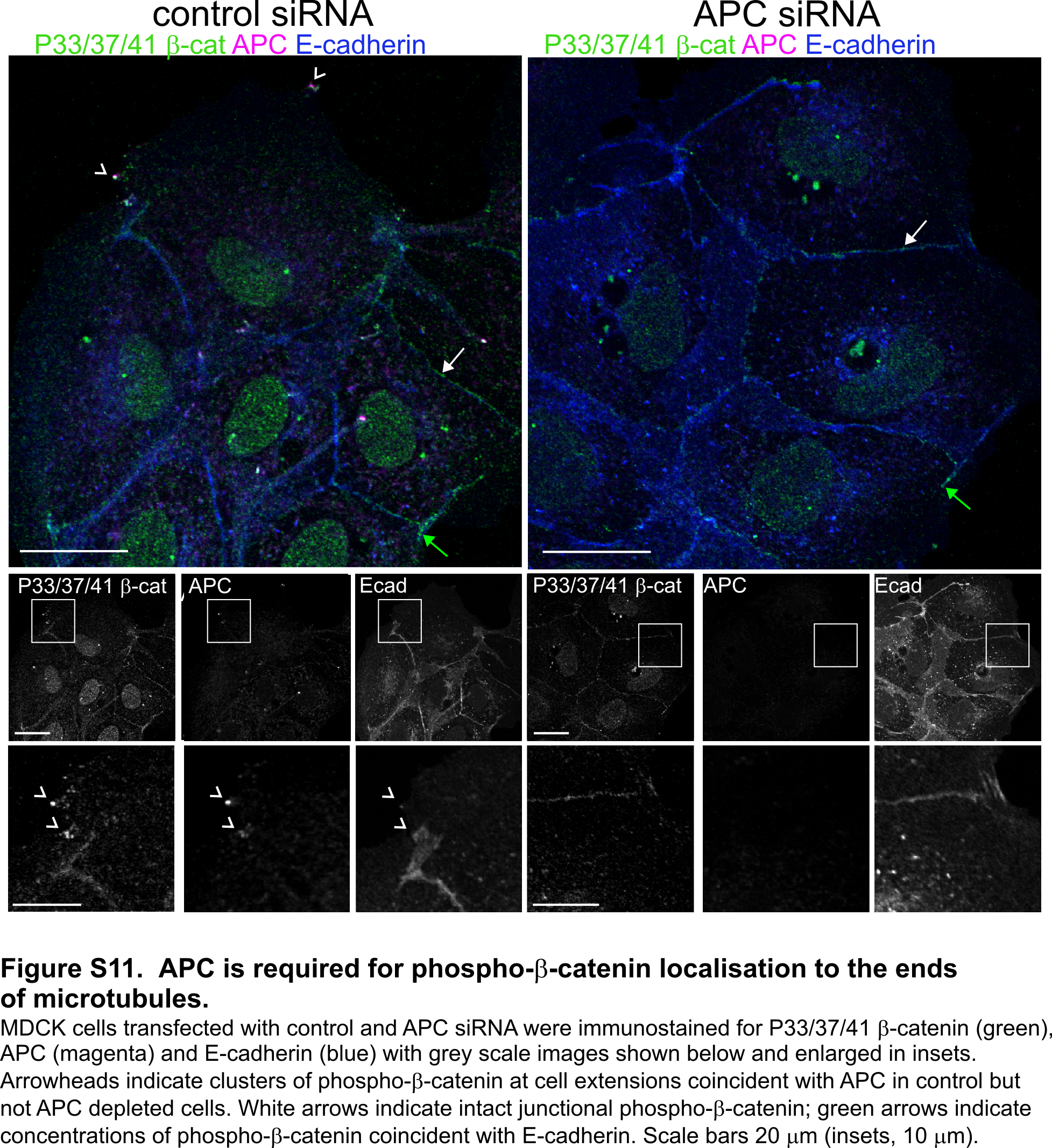

Supplement: Figure S11 — APC is required for phospho-β-catenin localisation to the ends of microtubules. MDCK cells transfected with control and APC siRNA were immunostained for P33/37/41 β-catenin (green), APC (magenta) and E-cadherin (blue) with grey scale images shown below and enlarged in insets. Arrowheads indicate clusters of phospho-β-catenin at cell extensions coincident with APC in control but not APC depleted cells. White arrows indicate intact junctional phospho-β-catenin; green arrows indicate concentrations of phospho-β-catenin coincident with E-cadherin. Scale bars 20 µ (insets, 10 µ). (5.37 MB TIF) [file pone.0014127.s011.tif]
